# Camrelizumab plus gemcitabine and oxaliplatin for relapsed or refractory classical Hodgkin lymphoma: a phase II trial

**DOI:** 10.1186/s12916-024-03329-8

**Published:** 2024-03-07

**Authors:** Yanfei Liu, Lingyan Ping, Yuqin Song, Yongjing Tang, Wen Zheng, Weiping Liu, Zhitao Ying, Chen Zhang, Meng Wu, Feier Feng, Ningjing Lin, Meifeng Tu, Jun Zhu, Yan Xie

**Affiliations:** Key Laboratory of Carcinogenesis and Translational Research (Ministry of Education), Department of Lymphoma, Peking University Cancer Hospital and Institute, Fucheng Road 52, Haidian Region, Beijing, 100142 China

**Keywords:** Hodgkin disease, Camrelizumab, Gemcitabine, Oxaliplatin

## Abstract

**Background:**

Classical Hodgkin lymphoma (cHL) is a highly curable disease, while novel therapy is needed for refractory or relapsed (R/R) patients. This phase II trial aimed to evaluate the role of camrelizumab plus gemcitabine and oxaliplatin (GEMOX) in R/R cHL patients.

**Methods:**

Transplant-eligible patients with R/R cHL were enrolled and received two 14-day cycles of camrelizumab 200 mg intravenously (IV) and two 28-day cycles of camrelizumab 200 mg IV, gemcitabine 1000 mg/m^2^ IV, and oxaliplatin 100 mg/m^2^ IV on days 1 and 15. Patients with partial response (PR) or stable disease received an additional cycle of combination therapy. Those who achieved complete response (CR) or PR proceeded to autologous stem cell transplantation (ASCT). The primary endpoint was the CR rate at the end of protocol therapy before ASCT.

**Results:**

Forty-two patients were enrolled. At the end of protocol therapy, the objective response rate and CR rate were 94.9% (37/39) and 69.2% (27/39) in the evaluable set, and 88.1% (37/42) and 64.3% (27/42) in the full analysis set, respectively. Twenty-nine patients (69.0%) proceeded to ASCT, and 4 of 5 patients with PR achieved CR after ASCT. After a median follow-up of 20.7 months, the 12-month progression-free survival rate was 96.6% and the 12-month overall survival rate was 100%. Grade 3 or higher treatment emergent adverse events occurred in 28.6% of patients (12/42), mainly hematological toxicity.

**Conclusions:**

Camrelizumab combined with GEMOX constitutes an effective salvage therapy for R/R cHL, proving to be relatively well-tolerated and facilitating ASCT in most patients, thus promoting sustained remission.

**Trial registration:**

ClinicalTrials.gov NCT04239170. Registered on January 1, 2020.

**Supplementary Information:**

The online version contains supplementary material available at 10.1186/s12916-024-03329-8.

## Background

Classical Hodgkin lymphoma (cHL) typically exhibits a favorable response to conventional treatments, leading to curative outcomes in a significant proportion of patients. However, an estimated 10 to 15% of patients display resistance to initial treatments, while nearly 30% experience a recurrence [1, 2]. For refractory or relapsed (R/R) cHL, the gold standard therapeutic approach is high-dose chemotherapy (HDCT) succeeded by autologous stem cell transplantation (ASCT). Previous research supports this strategy, revealing that a majority of R/R cHL patients achieve sustained remission post-ASCT [3, 4]. Importantly, individuals who secure a complete response (CR) prior to initiating HDCT and ASCT often have more favorable prognoses [5, 6]. Nonetheless, the effectiveness of traditional salvage chemotherapy regimens is variable. While they often result in high overall response rates (ORRs), the CR rates are relatively modest, highlighting the therapeutic challenges in this population.

The treatment landscape for R/R cHL has undergone marked transformation with the advent of novel therapeutic agents. Brentuximab vedotin (BV), utilized either as a standalone therapy or in combination with chemotherapy, has exhibited impressive response rates in the salvage therapy context [5, 7–10]. Yet, as BV’s usage proliferates in first-line treatment scenarios, it becomes less optimal for those presenting with R/R features. Venturing beyond BV, immune checkpoint inhibitors (ICIs) have risen to prominence as potent therapeutic tools, displaying impressive antitumor efficacy in R/R cHL. Consequently, they are now frequently employed as a pre-ASCT salvage regimen [11, 12]. A recent multicenter retrospective study suggests that ICI-based regimens, when used as pre-ASCT salvage strategies, provide enhanced event-free survival compared to traditional chemotherapy, BV–chemotherapy combinations, or BV alone [13]. Camrelizumab, a humanized IgG4 monoclonal antibody targeting PD-1, has illustrated exceptional efficacy with an ORR of 76% and a CR rate of 28% in Chinese R/R cHL patients, as evidenced in a phase II trial [14]. It is worth noting that the majority of this cohort consisted of patients either ineligible for ASCT or those who did not respond favorably to ASCT.

The therapeutic regimen of gemcitabine and oxaliplatin (GEMOX) has been conventionally deployed for patients with R/R cHL [15]. A distinguishing characteristic of GEMOX in the management of R/R cHL is its relatively diminished risk of myelosuppression and associated infections [15]. Furthermore, the lack of cross-resistance with frontline treatments underscores its potential clinical advantage. The combination of GEMOX with ICIs has shown to potentiate synergistic antitumor actions [16–18]. Given this promising backdrop, this study aimed to evaluate the therapeutic efficacy and safety of combining camrelizumab with the GEMOX chemotherapy regimen, followed by ASCT for patients with R/R cHL.

## Methods

### Study design and patients

In this phase II, single-arm, open-label study conducted at Peking University Cancer Hospital and Institute from March 2020 to July 2022, we enrolled patients aged 18 years and older with histologically confirmed R/R cHL and measurable lesions. Refractory cHL was characterized by an inability to attain a CR or partial response (PR) following the most recent treatment. Eligibility criteria encompassed a history of no more than three chemotherapy regimens, readiness for ASCT, an Eastern Cooperative Oncology Group performance status score between 0 and 1, a life expectancy exceeding 12 weeks, and satisfactory organ functionality. Exclusions pertained to those with active autoimmune diseases, active human immunodeficiency virus infections, untreated hepatitis B/C, a history of interstitial pneumonia, or prior exposure to PD-1/PD-L1 inhibitors or stem cell transplantation. Details of the eligibility criteria are outlined in the Additional file 1.

The study secured approval from Ethics Committee of Peking University Cancer Hospital and Institute and was executed in alignment with the Declaration of Helsinki and Good Clinical Practice guidelines. All participants provided informed consent. The trial is registered at ClinicalTrials.gov under the identifier NCT04239170.

### Procedure

Patients meeting eligibility criteria underwent treatment that commenced with two biweekly cycles of camrelizumab at a dose of 200 mg, administered intravenously (IV). This was followed by 4-week cycles in which camrelizumab (200 mg IV), gemcitabine (1000 mg/m^2^ IV), and oxaliplatin (100 mg/m^2^ IV) were delivered on the 1st and 15th days. Subsequent to this regimen, patients’ responses were evaluated. Those who achieved a CR progressed to ASCT. Conversely, individuals exhibiting progressive disease (PD) were discontinued from the study. Patients manifesting PR or a stable disease (SD) underwent an additional cycle of the combined treatment, followed by another response assessment. Achieving either CR or PR post-assessment qualified patients for ASCT. If the period spanning the protocol treatment and ASCT exceeded 4 weeks, supplementary doses of camrelizumab, up to two, were permissible. Given the reduced myelosuppression and infection risks inherent to the GEMOX regimen, the use of granulocyte colony-stimulating factors (G-CSF) and antibiotics for prophylaxis was not administered in this study.

Those attaining CR or PR were primed for stem cell mobilization, facilitated through G-CSF at a dosage of 10 µg/kg/day. For cases of suboptimal mobilization, plerixafor was employed based on the investigator’s judgment. Our center mandates a minimum transplant threshold of 1 × 10^6^/kg. Discretion regarding the conditioning for HDCT, ASCT, and post-transplant consolidation resided with the treating physician.

### Endpoints and assessments

The primary endpoint was the CR rate at the end of the protocol therapy before proceeding to ASCT. The secondary endpoints were ORR (CR plus PR), success rate of stem cell collection, progression-free survival (PFS, defined as the time from treatment initiation to disease progression or death), overall survival (OS, defined as the time from treatment initiation to death), and safety.

Response assessments were conducted using the Lugano Classification 2014 criteria. Fluorodeoxyglucose positron emission tomography/computed tomography (FDG-PET/CT) scans were undertaken at the following time points: baseline, after the second and third cycles of the camrelizumab plus GEMOX regimen, and 6 to 8 weeks post-ASCT, if applicable. Subsequent follow-up scans, either CT or PET-CT, were dictated by standard clinical practices. Adverse events (AEs) were characterized as per the Common Terminology Criteria for Adverse Events (CTCAE) v4.03. The definition of engraftment syndrome lacks standardization; within this study’s context, it was delineated as a noninfectious fever (≥ 38.0℃) accompanied by one or more of the following: skin rash, pulmonary infiltrates, or diarrhea.

### Statistical analysis

Simon’s two-stage design was adopted in this trial. Assuming that the null hypothesis of CR rate was 35% and the alternative hypothesis of CR rate was 55%, with a power of 80% and a one-side type I error of 0.05, 21 evaluable patients were required in the first stage. If more than 8 patients achieved CR, additional 18 evaluable patients were needed in the second stage. If more than 18 of 39 evaluable patients had CR, the treatment would be considered of further interest.

The full analysis set (FAS) comprised all patients who received at least one dose of the study drug. The evaluable set (ES) was a subset of the FAS and consisted of patients with at least one post-treatment response evaluation in the FAS. The efficacy analysis was conducted based on FAS and ES. The safety set (SS) was used for safety analyses and was defined as patients who received at least one dose of study drug.

To summarize baseline characteristics and toxicities, descriptive statistics were employed in this study. Categorical data were presented as frequencies and percentages. The 95% confidence intervals (CIs) for response rates were calculated using the Clopper–Pearson method. For time-to-event data, such as follow-up time, the median time was estimated using the Kaplan–Meier method, and the 95% CIs for the median time were calculated using the Brookmeyer–Crowley method. The PFS rate was estimated using the Kaplan–Meier method, and its 95% CI was calculated using the log–log method. All statistical analyses were conducted via SAS software, version 9.4 (SAS Institute).

## Results

### Patient characteristics

**Fig. 1:**
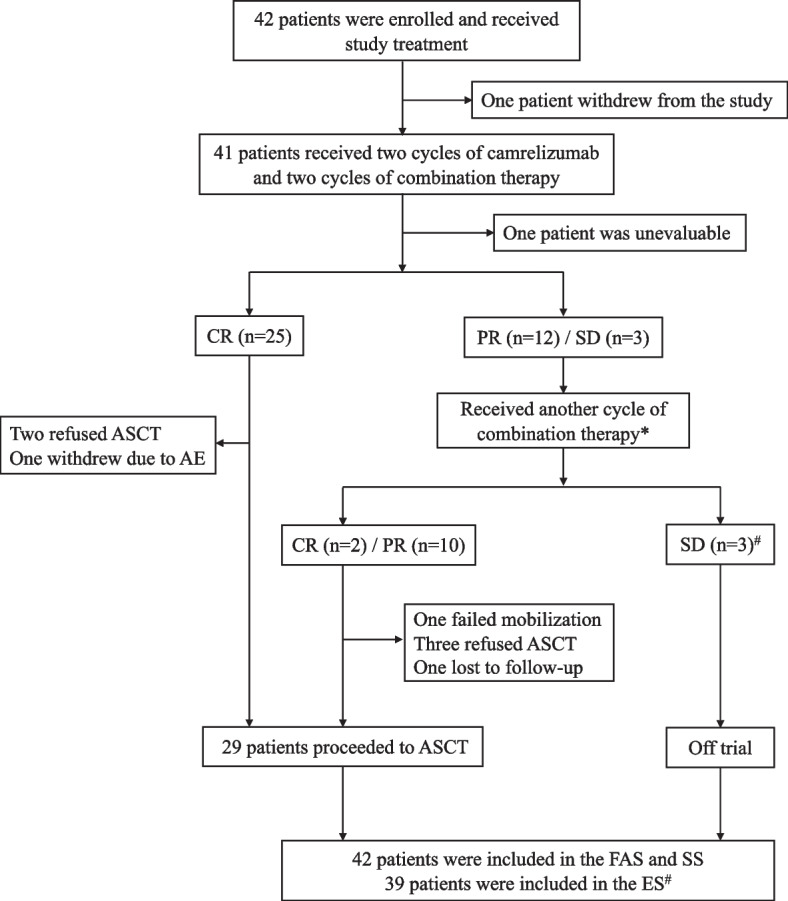
Patient flowchart. Asterisk indicates two patients did not receive another cycle of treatment due to COVID-19. Number sign indicates one participant was excluded from the ES due to the diagnosis of diffuse large B‐cell lymphoma found in the second biopsy. This patient initially presented with a large mediastinal mass and was refractory to first-line therapy. After 5 cycles of study treatment, the patient had stable disease according to PET-CT. However, a second mediastinal mass needle biopsy was performed and revealed diffuse large B-cell lymphoma (primary mediastinal). Upon review of the patient’s baseline biopsy, the pathological findings indicated the possibility of gray-zone lymphoma. The patient subsequently received 4 cycles of R-DA-EPOCH (rituximab, etoposide, prednisone, vincristine, cyclophosphamide, and doxorubicin) and radiotherapy. However, the disease was poorly controlled, and the patient developed adrenal invasion. CR complete response, PR partial response, SD stable disease, ASCT autologous stem cell transplantation, AE adverse event, FAS full analysis set, SS safety set, ES evaluable set

From March 2020 to July 2022, 42 patients were enrolled and received study therapy. All patients were included in the FAS and SS. One patient withdrew from the study after receiving two doses of camrelizumab due to personal reasons. Of the remaining 41 patients, all received at least 4 cycles of treatment, and 40 underwent a response assessment. One patient was excluded from the efficacy analysis due to a second biopsy revealing a diagnosis of diffuse large B-cell lymphoma, as described in the footnote of Fig. 1. Therefore, 39 patients were included in the ES.

Table 1 displays the baseline characteristics of all patients. The median age of the patients was 34 years (range, 21–58), and 66.7% of patients were male. The predominant pathologic subtype was nodular sclerosis (78.6%, 33/42). Twenty-six patients (61.9%) were at stage Ann Arbor III or IV. Primary refractory diseases were observed in half of the patients.

**Table 1:** Baseline characteristics of patients

Variables	All (*n* = 42)
Age, years, median (range)	34 (21–58)
Male sex, *n* (%)	28 (66.7)
ECOG PS score, *n* (%)	
0	41 (97.6)
1	1 (2.4)
Ann Arbor stage at time of enrolment, *n* (%)	
II	16 (38.1)
III	7 (16.7)
IV	19 (45.2)
Extranodal involvement Extranodal involvement, *n* (%)	23 (54.8)
Histologic subtype, *n* (%)	
Nodular sclerosis cHL	33 (78.6)
Mixed cellularity cHL	7 (16.7)
Lymphocyte-rich cHL	2 (4.8)
B symptoms, *n* (%)	14 (33.3)
Prior treatment line, *n* (%)	
1	37 (88.1)
2	5 (11.9)
First-line regimen, *n* (%)	
ABVD	38 (90.5)
ABVD + BEACOPP	2 (4.8)
ABVD + AVDP	1 (2.4)
ABVD + CHOPE	1 (2.4)
Second-line regimen (*n* = 5), *n* (%)	
DICE	3 (60.0)
CHOP	1 (20.0)
GDPE	1 (20.0)
Prior radiotherapy, *n* (%)	6 (14.3)
Disease status, *n* (%)	
Refractory	21 (50.0)
Relapsed within 1 year	9 (21.4)
Relapsed after 1 year	12 (28.6)

*ECOG PS* Eastern Cooperative Oncology Group performance status, *cHL* Classical Hodgkin lymphoma, *ABVD* Doxorubicin, bleomycin, vinblastine and dacarbazine, *BEACOPP* Bleomycin, etoposide, doxorubicin, cyclophosphamide, vincristine, procarbazine and prednisone, *AVDP* Doxorubicin, vinblastine, dacarbazine, and prednisone, *CHOPE* Cyclophosphamide, doxorubicin, vincristine, prednisone and etoposide, *DICE* Dexamethasone, ifosfamide, cisplatin, and etoposide, *CHOP* Cyclophosphamide, doxorubicin, vincristine, and prednisone, *GDPE* Gemcitabine, dexamethasone, cisplatin, and etoposide

### Treatment response

Following 4 cycles of therapy, the ORR was 88.1% (37/42) and the CR rate was 59.5% (25/42) in FAS. For 15 patients with PR or SD, an additional cycle of camrelizumab plus GEMOX was administered, resulting in an improvement of two PRs to CRs. Thus, at the completion of the protocol therapy, the ORR and CR rate were 94.9% (37/39) and 69.2% (27/39) in ES and were 88.1% (37/42) and 64.3% (27/42) in FAS, respectively (Table 2). The ORR and CR rate for patients with refractory disease were 90.5% (19/21) and 71.4% (15/21), respectively (Additional file 2: Fig. S1). Of the two patients who had SD, one received local radiotherapy and the other received BV monotherapy followed by ASCT, both of whom had sustained remission.

**Table 2:** Tumor response at the end of protocol therapy

Response	FAS (*n* = 42)	ES (*n* = 39)
ORR, *n* (%)	37 (88.1)	37 (94.9)
95%CI	74.4–96.0%	82.7–99.4%
CR, *n* (%)	27 (64.3)	27 (69.2)
95%CI	48.0–78.4%	52.4–83.0%
PR, *n* (%)	10 (23.8)	10 (25.6)
SD, *n* (%)	3 (7.1)	2 (5.1)
NE, *n* (%)	2 (4.8)	–

*FAS* Full analysis set, *ES* Evaluable set, *ORR* Objective response rate, *CI* Confidence interval, *CR* Complete response, *PR* Partial response, *SD* Stable disease, *NE* Not evaluable

### Stem cell collection and autologous stem cell transplantation

Seven patients who achieved CR or PR did not proceed to ASCT (Fig. 1). Thirty patients were administered mobilization regimens, with 90% (27/30) receiving G-CSF alone and 10% (3/30) receiving G-CSF plus plerixafor. One patient gave up stem cell collection due to failure of G-CSF mobilization. Twenty-nine patients underwent stem cell collection for 2–3 days, and the median count of harvested CD34^+^ cells per kg was 2.54 × 10^6^/kg (range, 1.02–7.94). A total of 86% (25/29) patients received additional camrelizumab before ASCT. At the last follow-up, 29 patients had undergone ASCT, with 24 achieving CR and 5 achieving PR at the time of ASCT. Four patients with PR achieved CR after ASCT. The median time to neutrophil and platelet engraftment were 11 days (range, 9–21) and 14 days (range, 8–30), respectively. Only one patient received one dose of camrelizumab consolidation therapy after transplantation.

### Survival

**Fig. 2:**
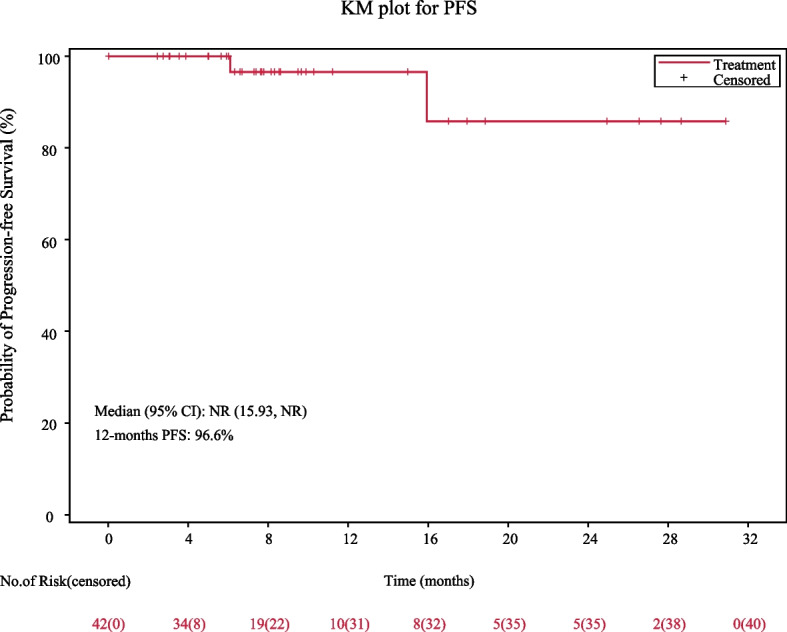
Kaplan‒Meier curve of progression-free survival (PFS). NR not reached, CI confidence interval

At the cutoff date of March 22, 2023, the median follow-up was 20.7 months (95%CI, 17.2–23.2), while the median follow-up following ASCT was 14.8 months (95%CI, 6.7–22.2). All patients are alive, and two experienced disease progression. None of the 29 patients who underwent ASCT experienced disease progression post-transplant. The 12-month PFS rate was 96.6% (95%CI, 77.9–99.5), and both median PFS and OS have not been reached (Fig. 2).

### Safety

Treatment emergent adverse events (TEAEs) and treatment-related adverse events (TRAEs) were reported in 41 and 40 patients (97.6% and 95.2%), respectively (Table 3, Additional file 2: Table S1). The most common TEAEs were alanine aminotransferase increased (24, 57.1%), followed by neutrophil count decreased (20, 47.6%). Grade 3 TEAEs occurred in 28.6% of patients (12/42), mainly hematological toxicities. Only one patient experienced grade 4 TEAEs (white blood cell decreased). The most common immune-related AE was alanine aminotransferase increased (38.1%). Six patients (14.3%) developed reactive cutaneous capillary endothelial proliferation, all of which were grade 1. Two patients had hypothyroidism; one had hyperthyroidism and one experienced thyroid stimulating hormone increased. Additionally, three patients with interstitial pneumonia were classified as immune-related.

**Table 3:** Treatment emergent adverse events (TEAEs) occurring at least 5% of patients

Events, *n* (%)	All	Grade 1	Grade 2	Grade 3	Grade 4
At least one TEAE	41 (97.6)	7 (16.7)	21 (50.0)	12 (28.6)	1 (2.4)
ALT increased	24 (57.1)	22 (52.4)	1 (2.4)	1 (2.4)	0
Neutrophil count decreased	20 (47.6)	6 (14.3)	8 (19.0)	6 (14.3)	0
Vomiting	19 (45.2)	10 (23.8)	8 (19.0)	1 (2.4)	0
Nausea	18 (42.9)	17 (40.5)	1 (2.4)	0	0
White blood cell decreased	16 (38.1)	4 (9.5)	9 (21.4)	2 (4.8)	1 (2.4)
AST increased	15 (35.7)	13 (31.0)	2 (4.8)	0	0
RCCEP	15 (35.7)	15 (35.7)	0	0	0
Hypertriglyceridemia	15 (35.7)	13 (31.0)	1 (2.4)	1 (2.4)	0
Platelet count decreased	15 (35.7)	11 (26.2)	2 (4.8)	2 (4.8)	0
Hyperuricemia	6 (14.3)	6 (14.3)	0	0	0
LDH increased	5 (11.9)	5 (11.9)	0	0	0
Anemia	5 (11.9)	5 (11.9)	0	0	0
Fever	4 (9.5)	4 (9.5)	0	0	0
Infectious pneumonia	4 (9.5)	0	4 (9.5)	0	0
Interstitial pneumonia	4 (9.5)	0	2 (4.8)	2 (4.8)	0
Pruritus	4 (9.5)	3 (7.1)	1 (2.4)	0	0
Anorexia	4 (9.5)	4 (9.5)	0	0	0
Hypokalemia	3 (7.1)	3 (7.1)	0	0	0

*ALT* Alanine aminotransferase, *AST* Aspartate aminotransferase, *RCCEP* Reactive cutaneous capillary endothelial proliferation, *LDH* Lactate dehydrogenase

Five patients experienced serious AEs. One patient had a fever after receiving the first dose of camrelizumab, which prolonged the hospital stay but did not affect the subsequent study treatment. Three patients developed interstitial pneumonia after 3 or 4 cycles of treatment, and all of them recovered after treatment interruption and corticosteroid therapy. One patient experienced infectious pneumonia and improved with symptomatic therapy. No deaths related to the treatment were reported. Six patients experienced dose delays due to TEAE (three due to aminotransferase increased, two due to interstitial pneumonia and one due to infectious pneumonia). Two patients had camrelizumab discontinuation due to interstitial pneumonia.

During ASCT, 22 patients (75.9%) developed febrile neutropenia. Ten patients (34.5%) experienced engraftment syndrome, which was mainly characterized by noninfectious fever and diarrhea (Additional file 2: Table S2). One patient exhibited fever and noncardiogenic pulmonary edema, but the symptoms were relieved with corticosteroid treatment. A 25-year-old female with no significant past medical or familial history experienced acute myocardial infarction and cardiogenic shock approximately 2 h post stem-cell infusion. She demonstrated rapid improvement following symptomatic treatment. No ASCT-related death was reported.

## Discussion

In the management of R/R cHL, HDCT followed by ASCT has traditionally been the cornerstone of treatment. Importantly, those patients who achieve a CR prior to ASCT often manifested more promising clinical outcomes [5, 6]. Although conventional salvage chemotherapy regimens boast commendable ORRs, their proficiency in achieving CR remains somewhat limited, underscoring the intricate challenges of treating this demographic. Our phase II data underscore the potential of combining camrelizumab with GEMOX, offering a therapeutic strategy that is both efficacious and well-tolerated for R/R cHL. This combined regimen not only facilitates a larger subset of patients to be candidates for ASCT but also amplifies the potential for sustained remission. In our cohort, the combined therapy yielded an ORR of 94.9% and a CR rate of 69.2%. Encouragingly, the durability of these remissions was evident, with no relapses reported among those who underwent ASCT.

The combination of camrelizumab and GEMOX for R/R cHL was chosen due to the lower risk of myelosuppression and infection associated with GEMOX and lack of cross-resistance with first-line treatments. GEMOX also demonstrated synergistic antitumor effects with ICIs. Gemcitabine, a nucleoside analog frequently used as a salvage treatment for HL, was found to increase MHC-I expression in tumor cells, leading to increased T-cell killing [16]. Additionally, gemcitabine selectively reduces the number of myeloid-derived suppressor cells (MDSCs), thus enhancing the immune system [17]. Besides, oxaliplatin can improve the function of dendritic cells (DCs) and enhance immune stimulation [16]. Oxaliplatin may also affect the immunosuppressive environment of tumors by reducing tumor cells’ expression of PD-L2 and enhancing T-cell recognition of tumor cells [18]. Furthermore, in vitro studies have found that chemotherapeutic drugs can induce the expression of PD-L1 in tumor cell lines, leading to tumor resistance, but blocking the PD-1/PD-L1 pathway can reverse drug resistance [19–21]. Anti-PD-1 therapy may re-sensitize tumor cells to chemotherapy for highly pretreated or primary refractory HL patients [22, 23]. In this study, where half of the patients were primary refractory to the latest chemotherapy and more than 20% of patients relapsed within 1 year, the combination of PD-1 inhibitor and chemotherapy resulted in a high remission rate.

In the current therapeutic landscape for R/R cHL, there has been a concerted effort to augment the efficacy of pre-ASCT treatments by using novel agents, either as monotherapies or in combination. For instance, as a primary salvage therapy, BV monotherapy yielded a CR rate of 27% which increased to 76% when sequentially combined with ifosfamide, carboplatin, and etoposide (ICE) [5]. The combination of BV and ICE presented CR and ORR rates of 69.2% and 94.8%, respectively [7]. Similar efficacies have been documented with BV when combined with alternate chemotherapy regimens [8–10]. Furthermore, a phase I–II study spotlighted the combination of BV and nivolumab, showcasing an impressive ORR of 85% and a CR rate of 67% as the first salvage therapy [24]. However, the ECHELON-1 trial’s success has shifted BV towards frontline therapy for advanced cHL [25], and its efficacy in early-stage HL, when combined with doxorubicin, vinblastine, and dacarbazine (AVD), has been highlighted [26]. This shift potentially curtails its utility in salvage therapy. Moreover, its high financial burden is a consideration in developing countries. The antitumor activity of PD-1 inhibitors in R/R cHL has been demonstrated, and their application as primary salvage therapies, preceding ASCT, is gaining attention. A phase II study depicted a synergistic effect when pembrolizumab was combined with gemcitabine, vinorelbine, and liposomal doxorubicin (GVD), achieving a high response rate, with 95% of participants progressing to ASCT. Furthermore, post-ASCT, 33% of patients received BV maintenance and remained in remission at the end of follow-up [11]. Another study underscored the safety and efficacy of pembrolizumab plus ICE chemotherapy for R/R cHL patients eligible for transplantation [27]. Additionally, employing nivolumab, either as a standalone or sequentially with ICE, yielded a promising ORR of 93% and a CR rate of 91%, with a successful bridge to ASCT for 79% of participants and a 2-year PFS of 94% [12]. Our study achieved a lower response rate compared to the pembrolizumab–GVD protocol. This difference may be attributed to the inclusion of patients with more refractory disease. It is crucial to note that the aforementioned studies predominantly encompassed patients who had undergone only one prior treatment, while our study included patients who failed two prior lines of treatment. These factors, coupled with the small sample size and the varied patient histories, underscore the need for cautious interpretation of the comparative efficacy and toxicity. Despite these challenges, our findings suggest that chemotherapy and immunotherapy might remain a potent strategy for those with prior treatments, although further validation is imperative.

The advent of novel agents has prompted a re-evaluation of the role of ASCT in treating R/R cHL. According to a previous study, patients with R/R cHL who achieved CR and discontinued anti-PD1 therapy had a 2-year PFS rate of 63%, indicating the potential for cure in some patients [28]. A recent phase II study assessed the use of tislelizumab combined with GEMOX for 6–8 cycles followed by tislelizumab maintenance for 2 years to treat R/R cHL, resulting in a 12-month PFS rate without ASCT of 96%, while the long-term survival outcome is pending [29]. Despite the high ORR observed in our study with combination therapy, it still appears necessary to perform ASCT, as neither of the patients who received ASCT relapsed in our study. However, long-term follow-up is needed.

The combination therapy demonstrated good tolerability, with no unexpected AEs observed. However, the incidence of engraftment syndrome appears to be higher than expected. Studies have demonstrated that previous anti-PD-1 therapy is an independent risk factor for engraftment syndrome. In a retrospective analysis, the incidence of engraftment syndrome was significantly higher in patients who received prior anti-PD-1 therapy than in those who did not (77.4% vs. 33.4%) [30]. A recent study evaluated the feasibility of ASCT after anti-PD1 treatment of R/R cHL, which showed similar toxicity profiles to previous ASCT data without anti-PD1 treatment, but two patients developed grade 4–5 autoimmune toxic myocarditis [31]. Since there are no standardized diagnostic criteria for engraftment syndrome, its rates may vary between studies. In our study, 34.5% of patients experienced engraftment syndrome, with most of them manifesting as noninfectious fever and diarrhea. Notably, our diagnostic approach primarily referenced the criteria established by Patel et al., and when applying the criteria of Maiolino et al. and Spitzer et al., a smaller subset of our patient cohort met these criteria, five patients (17.2%) and one patient (3.4%), respectively [32, 33]. This highlights the variability in diagnostic thresholds and underscores the importance of considering different criteria sets in the clinical assessment of engraftment syndrome. One patient in our study developed noncardiogenic pulmonary edema, which improved after corticosteroid treatment. Another patient experienced an acute myocardial infarction on the day of stem cell infusion, which was deemed unlikely to be related to immunotherapy. No transplant-related deaths occurred, and neutrophil and platelet engraftment was satisfactory. Patients receiving treatment present an elevated risk for engraftment syndrome, making timely intervention crucial. Furthermore, a thorough assessment of the patient’s overall health and pertinent risk factors, especially those linked to bleeding and infection, is essential to devise tailored management strategies.

One limitation of this study is the absence of data on patients treated with BV as first-line therapy or maintenance therapy post-ASCT, primarily due to its limited accessibility and the economic considerations prior to its inclusion in national insurance coverage. Nevertheless, previous studies have indicated that the incorporation of BV in the first-line setting does not seem to adversely affect the efficacy of subsequent immunotherapy and chemotherapy regimens [11]. Importantly, it must be underscored that the current study was conducted within a single institution in China, encompassing a relatively modest cohort of patients. This inherently constrains the diversity of the patient population and may limit the extrapolation of our findings to broader clinical settings. An important limitation of our study is the higher proportion of censored data, largely due to the COVID-19 pandemic disrupting routine clinical follow-ups. Besides, the follow-up duration in our study was relatively short. These factors potentially impact the robustness and completeness of our survival data. To address these limitations, future prospective multicenter studies with larger sample sizes are necessary. Furthermore, the long-term benefit of the regimen was unknown, and the follow-up is ongoing.

## Conclusions

In summary, camrelizumab plus GEMOX represents an effective salvage therapy for R/R cHL, with a tolerability profile that is relatively acceptable within this clinical context. Most patients were able to undergo ASCT and achieve sustained remission. However, further investigation is required to assess the long-term efficacy of the treatment and identify the patients who are most likely to derive benefit.

### Supplementary Information

**Additional file 1.** Study protocol.

**Additional file 2: Figure S1.** Subgroup analysis of complete response rate at the end of protocol therapy. **Table S1.** Treatment-related adverse events occurring at least 5% of patients. **Table S2.** Characteristics and management of engraftment syndrome.

## Data Availability

The data that support the findings of this study are available from the corresponding author upon reasonable request.

## Abbreviations

AE: Adverse event
ASCT: Autologous stem cell transplantation
AVD: Doxorubicin, vinblastine, and dacarbazine
BV: Brentuximab vedotin
cHL: Classical Hodgkin lymphoma
CI: Confidence interval
CR: Complete response
CTCAE: Common Terminology Criteria for Adverse Events
DC: Dendritic cell
ES: Evaluable set
FAS: Full analysis set
FDG-PET/CT: Fluorodeoxyglucose positron emission tomography/computed tomography
G-CSF: Granulocyte colony-stimulating factor
GEMOX: Gemcitabine and oxaliplatin
GVD: Gemcitabine, vinorelbine, and liposomal doxorubicin
HDCT: High-dose chemotherapy
ICE: Ifosfamide, carboplatin, and etoposide
ICI: Immune checkpoint inhibitor
IV: Intravenously
MDSC: Myeloid-derived suppressor cell
ORR: Overall response rate
OS: Overall survival
PD: Progressive disease
PFS: Progression-free survival
PR: Partial response
R/R: Refractory or relapsed
SD: Stable disease
SS: Safety set
TEAE: Treatment emergent adverse event
TRAE: Treatment-related adverse event
