# Supplementary material for: Camrelizumab plus gemcitabine and oxaliplatin for relapsed or refractory classical Hodgkin lymphoma: a phase II trial
Source: BMC Med. 2024 Mar 7;22:107. doi: 10.1186/s12916-024-03329-8 (PMC10921783; doi:10.1186/s12916-024-03329-8)
Supplement: Supplementary file 1 — Additional file 1. Study protocol. [file 12916_2024_3329_MOESM1_ESM.docx]

**An Open-label, Single-arm, Phase 2 Clinical Study of PD-1 Monoclonal Antibody Camrelizumab** **Combined with GEMOX in Patients with Relapsed or Refractory Classical Hodgkin Lymphoma Who Prepare to Receive Autologous Hematopoietic Stem Cell Transplantation**

# Table of Contents

[Table of Contents 2](#_Toc132123226)

[1.1 Study Background 4](#_Toc132123227)

[1.2 Scientific Rationale 5](#_Toc132123228)

[1.2.1 Study Rationale 5](#_Toc132123229)

[1.2.2 Rationale for Drug Development 5](#_Toc132123230)

[1.2.3 Rationale for Dosing Regimen Design 5](#_Toc132123231)

[1.3 Potential Risks and Benefits 6](#_Toc132123232)

[2. Study Objectives and Study Endpoints 7](#_Toc132123233)

[2.1 Study Objectives 7](#_Toc132123234)

[2.1.1 Primary Study Objective 7](#_Toc132123235)

[2.1.2 Secondary Study Objectives 7](#_Toc132123236)

[2.2 Study Endpoints 7](#_Toc132123237)

[2.2.1 Primary Study Endpoint 7](#_Toc132123238)

[2.2.2 Secondary Study Endpoints 7](#_Toc132123239)

[3. Study Design 7](#_Toc132123240)

[4. Selection and Withdrawal of Subjects 8](#_Toc132123241)

[4.1 Inclusion Criteria 8](#_Toc132123242)

[4.2 Exclusion Criteria 9](#_Toc132123243)

[4.3 Withdrawal Criteria 11](#_Toc132123244)

[4.4 Criteria for Discontinuation of Study Treatment 11](#_Toc132123245)

[4.5 Steps for Withdrawal from Study or Discontinuation of Study Treatment 12](#_Toc132123246)

[4.6 Subject Re-screening Criteria 12](#_Toc132123247)

[4.7 Premature Termination or Suspension of the Study 12](#_Toc132123248)

[5. Study Medication 13](#_Toc132123249)

[5.1 Overview of Study Drug 13](#_Toc132123250)

[5.1.1 Supply of Study Drug 13](#_Toc132123251)

[5.1.2 Dosage Form, Packaging and Labeling of Study Drug 13](#_Toc132123252)

[5.1.3 Storage and Stability of Study Drug 13](#_Toc132123253)

[5.1.4 Preparation of Study Drug 14](#_Toc132123254)

[5.1.5 Method of Use of Study Drug 14](#_Toc132123255)

[5.1.6 Dose Modification and Dose Delay 14](#_Toc132123256)

[5.2 Management, Dispensing and Recovery of Study Drug 18](#_Toc132123257)

[5.2.1 Destruction of Study Drug 18](#_Toc132123258)

[5.3 Concomitant Therapy 18](#_Toc132123259)

[5.3.1 Medications Used with Caution or Prohibited during the Study 18](#_Toc132123260)

[5.3.2 Permitted Concomitant Medications 19](#_Toc132123261)

[5.4 Management Recommendations for Immunology-related Adverse Events 19](#_Toc132123262)

[6. Study Procedures 22](#_Toc132123263)

[6.1 Screening Period 22](#_Toc132123264)

[6.2 Treatment Period 24](#_Toc132123265)

[6.3 End of Treatment 25](#_Toc132123266)

[6.4 Follow-up Period 26](#_Toc132123267)

[6.5 Unscheduled Visits 27](#_Toc132123268)

[6.6 Criteria for Continuing Treatment after Progression 27](#_Toc132123269)

[7. Evaluation 28](#_Toc132123270)

[7.1 Efficacy Evaluation 28](#_Toc132123271)

[7.2 Safety Evaluation 29](#_Toc132123272)

[7.2.1 Safety Parameters 29](#_Toc132123273)

[8. Adverse Event Reporting 29](#_Toc132123274)

[8.1 Adverse Events (AEs) 29](#_Toc132123275)

[8.1.1 Definition of AEs 29](#_Toc132123276)

[8.1.2 Criteria for Severity Grading of AEs 29](#_Toc132123277)

[8.1.3 Determination of Relationship between AEs and Investigational Product 30](#_Toc132123278)

[8.2 Serious Adverse Events (SAEs) 30](#_Toc132123279)

[8.2.1 Definition of SAE 30](#_Toc132123280)

[8.2.2 Hospitalization 31](#_Toc132123281)

[8.2.3 Disease Progression 32](#_Toc132123282)

[8.2.4 Potential Drug-induced Liver Injury 32](#_Toc132123283)

[8.2.5 Other Anti-tumor Therapies 33](#_Toc132123284)

[8.2.6 SAE Reporting System 33](#_Toc132123285)

[8.2.7 Follow-up of AEs/SAEs 33](#_Toc132123286)

[8.3 Pregnancy 33](#_Toc132123287)

[8.4 AEs of Special Interest (AESI) 34](#_Toc132123288)

[9. Data Analysis/Statistical Methods 34](#_Toc132123289)

[9.1 Determination of Sample Size 34](#_Toc132123290)

[9.2 Analysis Populations 35](#_Toc132123291)

[9.3 Statistical Analysis Plan 35](#_Toc132123292)

[9.3.1 General Analysis 35](#_Toc132123293)

[9.3.2 Basic Analyses 35](#_Toc132123294)

[9.3.3 Analysis of Efficacy Endpoints 36](#_Toc132123295)

[9.3.4 Safety Analysis 36](#_Toc132123296)

[9.3.5 Interim Analysis 36](#_Toc132123297)

[10 Study Management 36](#_Toc132123298)

[10.1 Ethics and Informed Consent 36](#_Toc132123299)

[10.2 Amendments to the Protocol 37](#_Toc132123300)

[10.3 Quality Assurance of the Clinical Study 37](#_Toc132123301)

[10.4 Data Management 38](#_Toc132123302)

[10.4.1 Data Collection 38](#_Toc132123303)

[10.4.2 Data Management and Quality Control 39](#_Toc132123304)

[10.4.3 Review of Data and Monitoring of the Study Site 39](#_Toc132123305)

[10.4.4 Retention of Study Records 40](#_Toc132123306)

[11 Data Processing and Intellectual Property 40](#_Toc132123307)

[11.1 Data Processing 40](#_Toc132123308)

[11.2 Publication of Study Results 41](#_Toc132123309)

[12 Clinical Study Progress 41](#_Toc132123310)

1. Introduction: Study Background and Scientific Rationale

## 1.1 Study Background

Hodgkin lymphoma (HL) is a malignancy with a low incidence but good prognosis. It often occurs in young people, and most patients can achieve long-term response with standard chemoradiotherapy. According to WHO classification, there are two main types of HL: nodular lymphocyte-predominant Hodgkin lymphoma (NLPHL) and classical Hodgkin lymphoma (cHL). In western countries, cHL accounts for about 95%. The discussion in the text below is specific to cHL. Based on the modern comprehensive therapies, the 10-year overall survival (OS) is more than 90% in limited-stage cHL and the 5-year OS is more than 80% in advanced cHL. However, relapse or progression still occurs in 10% to 30% of patients. High dose chemotherapy (HDCT) combined with autologous stem cell transplantation (ASCT) is currently recommended for these patients after response to salvage therapy. Such treatment leads to long-term response in approximately 50% of patients.

Data from other investigators and our own data suggest that the progression-free survival (PFS) after transplantation is significantly better in patients who achieve complete response (CR) or have negative positron emission tomography with computed tomography (PET-CT) before transplantation than in those who achieve partial response (PR) and those having no response (PET-CT positive) before transplantation. Professor Adams et al. have reported the results of a meta-analysis. They collected data from 11 studies on autoSCT in 745 patients with relapsed or refractory cHL, all of whom underwent PET-CT before transplantation. It was found that in patients with positive PET-CT before transplantation, PFS ranged from 0% to 52% and OS ranged from 17% to 72%, whereas in patients with negative PET-CT before transplantation, PFS ranged from 55% to 85% and OS ranged from 78% to 100%. Therefore, if more patients can achieve CR or negative PET-CT after salvage therapy, they may have a better outcome after transplantation, thereby improving the overall effectiveness in patients with relapsed or refractory HL.

Traditional salvage chemotherapy regimens mainly include ICE, ESHAP, DHAP, GDP, GVD, etc., and the CR rate is basically 20% to 40%. Researchers have tried to use gemcitabine combined with bendamustine, GEMOX, etc. as salvage therapy in some other studies, and the CR rates are 33% and 38%, respectively. In addition, second-line salvage therapy with single-agent brentuximab vedotin (BV) or in combination with chemotherapy can even achieve a CR rate of 69% to 90%. However, brentuximab vedotin has not been marketed in China, therefore, it is not currently available for routine clinical use.

The effective rate of PD1 monoclonal antibodies in patients with relapsed or refractory HL is approximately 70% to 80%, and the CR rate is approximately 20% to 30%. However, these data are mostly derived from HL patients who relapse after transplantation. Therefore, their indications are HL that relapses after stem cell transplantation. Some studies are trying to use PD1 monoclonal antibodies in combination with chemotherapy for the salvage therapy of relapsed and refractory HL, but the results have not been published.

## 1.2 Scientific Rationale

### 1.2.1 Study Rationale

Programmed death-1 (PD-1), a protein receptor expressed on the surface of T cells, binds to programmed death-ligand 1 (PD-L1), which can conduct inhibitory signals and reduce the proliferation of T cells. Expression of PD-L1 is found in many human solid tumors, which is one of the reasons for their escape from immune killing. Monoclonal antibodies that block the PD-1/PD-L1 signaling pathway have shown good efficacy in a variety of solid tumors and hematological tumors and have been approved by FDA. Overexpression of PD-L1 is present in most of tumor tissues of patients with cHL, and the efficacy of anti-PD1 treatment is also the highest in available reports.

### 1.2.2 Rationale for Drug Development

Jiangsu Hengrui Pharmaceuticals Co., Ltd. (hereinafter referred to as "Hengrui Pharmaceuticals") has identified a series of PD-1 antibodies in mice using PD-1 as the target and PD-1 recombinant protein as the immunogen. The lead antibody was selected through a large number of in vitro binding experiments, in vitro ligand blocking experiments, T-cell proliferation experiments, animal experiments and antibody druggability experiments. Further, a computer-simulated humanized design was performed for the obtained murine lead antibody, and a number of humanized PD-1 monoclonal antibodies were synthesized, among which the antibody, camrelizumab (drug number: SHR-1210), with the highest activity was developed as the focus. Preclinical data suggest that camrelizumab has similar anti-tumor effects in vitro and in vivo to nivolumab and pembrolizumab (both are marketed drugs of the same class). Moreover, three Phase 1 clinical studies of camrelizumab have been conducted in Chinese patients with solid tumors, and the dose escalation has been completed. No dose-limiting toxicity is found. The maximum tolerated dose (MTD) has not been reached, and camrelizumab is well tolerated. This study aims to further explore the efficacy and safety of camrelizumab in combination with chemotherapy in potentially beneficial tumor type, cHL.

### 1.2.3 Rationale for Dosing Regimen Design

PD-1 immune checkpoint inhibitor achieves the purpose of contact-dependent immunosuppression by blocking PD-1/PD-L1 receptor binding. Therefore, the occupancy of the inhibitor to the PD-1 receptors is the underlying pharmacological mechanism for the ultimate anti-tumor effect. Results from the Phase 1 clinical pharmacokinetic study showed that the plasma concentration decreased to approximately 1000 ng/mL or less on Day 15 after single fixed doses of 1 mg/kg and 60 mg, and the plasma concentration was about 4000-9000 ng/mL on Day 15 after single fixed doses of 3 mg/kg and 200 mg, while the development of receptor occupancy studies suggested that the plasma concentration should be above 2000 ng/mL to maintain saturation of receptor occupancy. The actual clinical results also showed that the saturation of receptor occupancy could be maintained at all times at a fixed dose of 200 mg once every 2 weeks, and no positive correlation between the incidence of adverse events and the dose was observed yet in the Phase 1 study, whether administered by body weight or at a fixed dose. Integrating the above study observations and considering the convenience of clinical medication, a fixed dose of 200 mg once every 2 weeks is selected as the dosing method for this study.

Gemcitabine in combination with oxaliplatin (GEMOX) is a common chemotherapy regimen and is often used as the salvage regimen for lymphoma. Lots of clinical practice has proved that its efficacy is good and the toxic and side effects are controllable. Standard doses and intervals will be followed in this study.

## 1.3 Potential Risks and Benefits

Camrelizumab is a humanized PD-1 antibody independently developed by Hengrui Pharmaceuticals. Preclinical data suggest that camrelizumab has similar pharmacodynamic and anti-tumor effects to nivolumab and pembrolizumab. At present, the ongoing Phase 1 clinical study in China has found that it has also shown definite efficacy in solid tumors of different types and has entered the Phase 3 clinical study phase. GEMOX is the common salvage chemotherapy regimen with definite efficacy in patients with relapsed and refractory cHL. The possible benefit for subjects enrolled in this study is a higher rate of complete response, thereby improving the efficacy of ASCT. Treatment data from these subjects will make it possible to develop new salvage therapy patterns for relapsed and refractory cHL. The adverse events with a high incidence in the Phase 1 study of camrelizumab were mainly reactive cutaneous capillary endothelial proliferation, rash, fever, ALT increased, AST increased, and hypothyroidism, but the vast majority were Grade I-II and recovered with no impact on the continued use of the study drug. Common adverse events of GEMOX chemotherapy are mainly myelosuppression, gastrointestinal reactions, neurotoxicity, rash, fever, etc. In addition, adverse events such as local bleeding, infection, and allergy may also occur due to blood sampling and imaging examinations during treatment.

# 2. Study Objectives and Study Endpoints

## 2.1 Study Objectives

### 2.1.1 Primary Study Objective

To evaluate the complete response (CR) of PD-1 antibody camrelizumab combined with GEMOX in patients with relapsed or refractory classical Hodgkin lymphoma who prepare to receive autologous hematopoietic stem cell transplantation.

### 2.1.2 Secondary Study Objectives

- To observe and evaluate the objective response rate (ORR), stem cell harvest success rate, and PFS of PD-1 antibody camrelizumab combined with GEMOX in patients with relapsed or refractory classical Hodgkin lymphoma who prepare to receive autologous hematopoietic stem cell transplantation.
- To evaluate the safety of PD-1 antibody camrelizumab combined with GEMOX in patients with relapsed or refractory classical Hodgkin lymphoma who prepare to receive autologous hematopoietic stem cell transplantation.

## 2.2 Study Endpoints

### 2.2.1 Primary Study Endpoint

- Complete response (CR)

### 2.2.2 Secondary Study Endpoints

- Objective response rate (ORR)
- Stem cell harvest success rate
- Progression-free survival (PFS)
- Overall survival (OS)
- Safety: Adverse events (AEs), clinical laboratory data assessments

# 3. Study Design

This study will be conducted in an open-label, single-arm, single-center design to evaluate the efficacy and safety of camrelizumab combined with GEMOX in patients with relapsed or refractory classical Hodgkin lymphoma who prepare to receive autologous hematopoietic stem cell transplantation. Eligible subjects will be treated in two stages. Single-agent stage: camrelizumab: 200 mg/dose by IV infusion over 30 min (not less than 20 min and not more than 60 min, including the flush stage), once every other week (q2w) for a total of 2 doses, then all patients will enter the combined therapy stage: camrelizumab: 200 mg/dose by IV infusion on Day 1 and Day 15; gemcitabine: 1000 mg/m^2^ by IV injection on Day 1 and Day 15; oxaliplatin: 100 mg/m^2^ by IV injection on Day 1 and Day 15; every 28-day cycle. Response will be evaluated after 2 cycles of combined therapy. Patients with negative PET-CT (CR) will directly enter the autologous transplantation stage, and patients with positive PET-CT and PD will be directly withdrawn from the study. If PET-CT result is positive but the response is PR or SD, another cycle of combined therapy will be conducted for re-evaluation. Patients with negative PET-CT (CR) and patients with positive PET-CT but PR will enter the transplantation stage, and other patients will be withdrawn from the study. If the waiting time for the subject to receive transplantation after achieving CR or PR exceeds 4 weeks, 1-2 additional doses of camrelizumab monotherapy may be considered to ensure that the subject is in response. Patients may also be withdrawn from the study if they experience intolerable toxicity or other reasons specified in the protocol. Subjects who complete the treatment will enter the follow-up period for safety follow-up or survival follow-up.

# 4. Selection and Withdrawal of Subjects

## 4.1 Inclusion Criteria

Subjects who meet all of the following inclusion criteria may be included in this study.

1. Aged ≥ 18 years, male and female;
2. Histologically confirmed classical Hodgkin lymphoma;
3. Patients who have relapsed (disease progression confirmed after most recent treatment) or refractory (failure to achieve a complete response or partial response after most recent therapy) and who prepare to receive autologous hematopoietic stem cell transplantation. Patients should not receive more than three lines of chemotherapy, and are required to provide 10-15 unstained slides of tumor tissue;
4. Subjects must have measurable lesions. Measurable lesions are defined as the longest diameter of lymph node lesions in CT cross-sectional images > 1.5 cm; or the longest diameter of extranodal lesions > 1.0 cm;
5. The Eastern Cooperative Oncology Group (ECOG) performance status must be 0 or 1;
6. Life expectancy ≥ 12 weeks;
7. All laboratory tests at screening should be performed as required by the protocol and within 7 days prior to enrollment. The values of laboratory tests performed for screening must meet the following criteria:

**Hematology** (no blood transfusion, no use of granulocyte colony stimulating factor [G-CSF], no use of medication for abnormality correction within 14 days prior to screening):

1. Hemoglobin (Hb) ≥ 90 g/L;
2. Neutrophils (ANC) ≥ 1.5 × 10^9^/L;
3. Platelets (PLT) ≥ 100 × 10^9^/L;

**Blood chemistry:**

1. TBIL < 1.5× upper limit of normal (ULN);
2. Glutamate alanine aminotransferase (ALT) and glutamate aspartate aminotransferase (AST) ≤ 2.5 × ULN;
3. Serum creatinine (Cr) ≤ 1.25 × ULN or endogenous creatinine clearance ≥ 60 mL/min (Cockcroft-Gault formula);

**Coagulation** (unless the subject is receiving anticoagulant therapy, and coagulation parameters [PT/INR and APTT] are within the expected range of anticoagulant therapy at screening):

1. International normalized ratio (INR) ≤ 1.5 × ULN;
2. Activated partial thromboplastin time (APTT) ≤ 1.5 × ULN;
3. Women of childbearing potential must have a negative serum pregnancy test within 7 days prior to the first dose and be willing to use highly effective methods of contraception during the study and for 1 year after the last dose of the investigational product. Male subjects with female partners of childbearing potential should be surgically sterile or agree to use highly effective methods of contraception during the study and within 1 year after the last dose of investigational product.
4. Subjects who voluntarily join the study, sign the informed consent form (ICF), have good compliance, and cooperate with the follow-up.

## 4.2 Exclusion Criteria

Patients who meet any of the following criteria will be excluded from the study:

1. The pathological diagnosis was nodular lymphoid predominant Hodgkin lymphoma;
2. Medical history and concomitant diseases
3. Subject with any active, known, or suspected autoimmune disease. Subjects with stable disease and requiring no systemic treatment with immunosuppressive agents can be enrolled;
4. Subjects requiring systemic treatment with either corticosteroids (> 10 mg/day of prednisone or equivalent) or other immunosuppressive agents within 14 days prior to administration of study drug. Inhaled or topical steroids and adrenal hormone replacement therapy at doses > 10 mg/day prednisone equivalents are acceptable in the absence of active autoimmune disease;
5. Use of anti-tumor vaccines or other anti-tumor therapies with immunostimulatory effects within 3 months prior to administration of study drug;
6. Previous use of anti-PD-1 antibody, anti-PD-L1 antibody, anti-PD-L2 antibody, or anti-CTLA-4 antibody (or any other antibodies acting on T-cell costimulation or checkpoint pathway); or prior chemotherapy with GEMOX regimen and response evaluation of PD;
7. Patients who are participating in other clinical studies, or will receive the first dose of study drug with an interval of less than 4 weeks from the end of treatment in the previous clinical study;
8. Known history of interstitial pneumonia;
9. History of other malignancies; with the exception of patients with basal cell carcinoma of the skin, superficial bladder cancer, squamous cell carcinoma of skin, or cervix carcinoma in situ who have undergone potentially curative treatment and have not relapsed within 5 years of treatment initiation;
10. Patients who have previously received tumor chemotherapy, radiotherapy and immunotherapy, including those with an interval of less than 4 weeks from loco-regional therapy; patients with anti-tumor therapy-related adverse reactions (except alopecia) that have not recovered to NCI-CTCAE ≤ Grade 1 after prior systemic anti-tumor therapy;
11. Patients who have received autologous or allogeneic hematopoietic stem cell transplantation;
12. Patients who have undergone major surgery or severe trauma prior to enrollment, and the impact of the surgery or trauma has been eliminated for less than 28 days;
13. Patients with active pulmonary tuberculosis (TB) should be excluded. Subjects with suspected active TB should be excluded through tests of chest x-ray, sputum, clinical symptoms and signs. Patients with a history of active pulmonary tuberculosis infection within the previous year should be excluded even if they have been treated; patients with a history of active pulmonary tuberculosis infection more than 1 year ago should also be excluded unless the course and type of prior anti-tuberculosis therapy used are demonstrated to be appropriate;
14. Severe acute or chronic infection requiring systemic treatment;
15. Patients with hypertension that is not well controlled with antihypertensives (systolic blood pressure ≥ 140 mmHg or diastolic blood pressure ≥ 90 mmHg);
16. Patients with heart failure (New York Heart Association Criteria Class III or IV) and poor control of coronary artery disease or arrhythmia despite appropriate medical treatment, or a history of myocardial infarction within 6 months prior to screening;
17. History of clinically significant hemorrhage symptoms or significant hemorrhagic diathesis within 3 months prior to randomization, such as digestive tract hemorrhage, hemorrhagic gastric ulcer, fecal occult blood ++ or above at baseline, or vasculitis, etc.;
18. Physical examination and laboratory test findings
19. Known history of positive human immunodeficiency virus (HIV) test or known acquired immunodeficiency syndrome (AIDS);
20. Untreated active hepatitis (hepatitis B: hepatitis B surface antigen [HBsAg] positive and hepatitis B virus [HBV] deoxyribonucleic acid [DNA] ≥ 500 IU/mL; hepatitis C: hepatitis C virus [HCV] ribonucleic acid [RNA] positive and abnormal liver function); coinfection with hepatitis B and hepatitis C;
21. Patients who may have other factors that may lead to the forced discontinuation of the study, as assessed by the investigator, such as other serious diseases or serious laboratory abnormalities or other family or social factors that may affect the safety of the subjects, or the collection of study data and samples;
22. Pregnant or breastfeeding female patients.

## 4.3 Withdrawal Criteria

Reasons for subjects' withdrawal from the study may include:

1. The subject withdraws informed consent of participating in the study, and rejects further follow-up;
2. The patient experiences any clinical adverse reaction, laboratory abnormalities, or intercurrent illnesses, and the investigator believes that continued participation in the study is not in the best interest of the patient;
3. Other conditions in which withdrawal from the study is deemed necessary by the investigator, for instance, the subject losses the ability to express his/her will freely due to confinement or isolation;
4. Loss to follow-up;
5. Death of subject;
6. Study discontinued by sponsor.

## 4.4 Criteria for Discontinuation of Study Treatment

Subjects must discontinue study drug for any of the following circumstances:

1. The subject requests discontinuation of the study drug;
2. Disease progression is indicated by medical imaging or clinical features, unless the subject meets the criteria for continued treatment after disease progression;
3. The subject becomes pregnant during the study;
4. There are any clinical AEs, laboratory abnormalities, or other medical conditions resulting in that the subject may no longer benefit from continued treatment;
5. The subject is unable to continue the study due to overall deterioration of health status;
6. The subject is found ineligible or with other significant protocol deviations after enrollment;
7. Loss to follow-up;
8. Study discontinued by sponsor;
9. Death of subject;
10. Other reasons that lead to treatment discontinuation in the investigator's opinion.

## 4.5 Steps for Withdrawal from Study or Discontinuation of Study Treatment

Every effort must be made to complete the protocol-specified efficacy and safety tests at the time of withdrawal from the study and the safety follow-up period, and to completely document AEs and outcomes. The investigator may suggest or provide new or alternative treatments to the subject based on the actual situation of the subject. Subjects who withdraw from the study for reasons other than PD should be followed up for radiographic evaluation until the initiation of a new anti-tumor therapy or disease progression.

If a subject refuses to come to the study site for further visits, the status of his/her survival should continue to be followed up unless the subject withdraws consent to disclose further information or be contacted, in which cases, no further study evaluations should be performed and no further data should be collected.

## 4.6 Subject Re-screening Criteria

Re-screening is allowed in this study, i.e., subjects who enter the screening process but are not eligible for enrollment and do not start treatment may be re-enrolled. At re-screening, subjects must re-sign the ICF and obtain a new subject number.

## 4.7 Premature Termination or Suspension of the Study

The study may be prematurely terminated or suspended, if justified, possibly due to the decision of the regulatory authority, change in opinion of the Ethics Committee, the efficacy or safety concerns of the study drug, or the decision of the sponsor. The party deciding to suspend/terminate the study will issue a written notice and record the reason for the termination or suspension of the study to the investigator, the sponsor and the regulatory authority. The investigator should immediately inform the Ethics Committee and the sponsor and provide relevant reasons.

Reasons for premature termination or suspension of the study may include:

1. The study brings definite unexpected, major or unacceptable risks to subjects;
2. Existing efficacy results support premature termination of the study;
3. Compliance with protocol requirements is low;
4. The data is incomplete or unmeasurable;
5. The study results provides no utilization value.

If the above-mentioned drug safety, protocol compliance and data quality issues causing study suspension are resolved, the study can be continued with the consent from the sponsor, Ethics Committee or CFDA.

# 5. Study Medication

## 5.1 Overview of Study Drug

### 5.1.1 Supply of Study Drug

Camrelizumab is manufactured by Suzhou Suncadia Biopharmaceuticals Co., Ltd. Gemcitabine and oxaliplatin are prescribed directly from the Pharmacy of Beijing Cancer Hospital.

### 5.1.2 Dosage Form, Packaging and Labeling of Study Drug

Table 1 General Information of Study Drug

| Name | **Camrelizumab for Injection** |
| --- | --- |
| Manufacturer | Suzhou Suncadia Biopharmaceuticals Co., Ltd. |
| Appearance | White or off-white lyophilized powder |
| Route of administration | Intravenous injection |
| Strength | 200 mg/20 mL vial |
| Storage and stability | Store in a medical refrigerator at 2-8°C, with a shelf life of 2 years tentatively. This product should not be frozen. |

Labeling: Refer to the Pharmacy Manual, subject to the actual drug labeling.

Gemcitabine and oxaliplatin are subject to the actual drugs of Beijing Cancer Hospital.

Camrelizumab is used as the drug name in premarketing clinical studies of SHR-1210, and the two names refer to the same drug ^[36]^.

## 5.1.3 Storage and Stability of Study Drug

The investigator, or his/her authorized representative (e.g., pharmacist), will ensure that all study drugs are stored under required storage conditions in an access-controlled safe area in accordance with applicable regulatory requirements. The study drugs should be stored in accordance with the storage conditions listed in 5.1.2. If the storage conditions in the protocol is inconsistent with those in other materials, the storage conditions on the label should prevail for camrelizumab.

Once any deviation from the label is found, it should be promptly reported. The study site should take active measures to transfer the study drug to a place under specified storage conditions on the label as soon as possible, and report the temperature deviation and the measures taken to the sponsor.

The study drug affected by temperature deviations should be temporarily separated and should not be used until sponsor's permission (not regarded as a protocol deviation). Use of affected study drug without the sponsor's permission is a protocol deviation. The sponsor will provide the study site with the specific steps for reporting of temperature deviations.

### 5.1.4 Preparation of Study Drug

The camrelizumab, gemcitabine, and oxaliplatin used in this study are administered by intravenous drip. Therefore, the drug should be prepared by qualified or experienced study personnel such as study nurses. The investigational product, camrelizumab, does not contain preservatives, and aseptic operation should be strictly followed during the drug preparation. Drug preparation is detailed in the Pharmacy Manual.

## 5.1.5 Method of Use of Study Drug

Single-agent stage: camrelizumab: 200 mg/dose by IV infusion over 30 min (not less than 20 min and not more than 60 min, including the flush stage), once every other week (q2w) for a total of 2 doses. Combined therapy stage: camrelizumab: 200 mg/dose by IV infusion on Day 1 and Day 15; gemcitabine: 1000 mg/m^2^ by IV injection on Day 1 and Day 15; oxaliplatin: 100 mg/m^2^ by IV injection on Day 1 and Day 15; every 28-day cycle. Infusion of gemcitabine and oxaliplatin should be performed in accordance with routine clinical procedures. Doses in Cycle 2 and subsequent cycles may be given within 3 days before or 7 days after the day of scheduled dosing, and any time beyond this period will be considered a dose delay. The timing of subsequent doses should be calculated based on the actual date of the previous dose. Examinations and assessments for visit should be completed prior to each dose.

### 5.1.6 Dose Modification and Dose Delay

**Dose Modification of Camrelizumab**

Adverse events associated with camrelizumab may be immune-related AEs (irAEs) and may occur within a short period of time after the first dose, or several months after the last dose. If an event listed in the table below occurs, the dosing of camrelizumab should be withheld. If the measures listed in the table are considered inappropriate based on the benefit/risk ratio for the subject, or a situation not listed in the table is encountered in the course of clinical operation, which requires interruption or resume of camrelizumab dosing, it should be discussed with the sponsor before making a decision.

During the study, camrelizumab is allowed to be interrupted for a maximum of 12 weeks; if the dose of camrelizumab is delayed for more than 7 days after the scheduled dose time, the delayed dose will not be made up, and the 200 mg dose will be continued at the next scheduled dose time.

Table 2 Dose Modification Criteria of Camrelizumab

| **Drug-Related Immune-Related Adverse Event (irAE)** | **AE Grades Requiring Dose Interruption** | **Time for Restarting Treatment** | **Camrelizumab Treatment** |
| --- | --- | --- | --- |
| Diarrhea/Colitis | 2-3 | Recovery to Grade 0-1 | Treatment should be discontinued if AE is not resolved within 12 weeks after the last dose, or the corticosteroid dose cannot be reduced to ≤ 10 mg prednisone or its equivalent within 12 weeks. |
|  | 4 | Treatment discontinuation | Discontinue treatment |
| AST, ALT or bilirubin increased | 2 | Recovery to Grade 0-1 | Treatment should be discontinued if AE is not resolved within 12 weeks after the last dose. |
|  | 3-4 | Treatment discontinuation | Discontinue treatment |
| Hyperthyroidism | 3 | Recovery to Grade 0-1 | Treatment should be discontinued if AE is not resolved within 12 weeks after the last dose, or the corticosteroid dose cannot be reduced to ≤ 10 mg prednisone or its equivalent within 12 weeks. |
|  | 4 | Treatment discontinuation | Discontinue treatment |
| Hypothyroidism |  | Treatment may be resumed after initiation of thyroid hormone replacement therapy | Treatment may be resumed after initiation of thyroid hormone replacement therapy |
| Pneumonitis | 2 | Recovery to Grade 0-1 | Treatment should be discontinued if AE is not resolved within 12 weeks after the last dose, or the corticosteroid dose cannot be reduced to ≤ 10 mg prednisone or its equivalent within 12 weeks. |
|  | 3-4 | Treatment discontinuation | Discontinue treatment |
| Immune-related hypophysitis | 2-3 | Recovery to Grade 0 or 1; camrelizumab treatment may be resumed after initiation of endocrine replacement therapy | Treatment should be discontinued if AE is not resolved within 12 weeks after the last dose, or the corticosteroid dose cannot be reduced to ≤ 10 mg prednisone or its equivalent within 12 weeks. |
| Type I diabetes mellitus (new onset) or hyperglycemia | New onset of type I diabetes or Grade 3-4 hyperglycemia with evidence of β-cell depletion | After clinical and metabolic stabilization | Continue treatment with camrelizumab. |
| Renal failure or nephritis | 2 | Recovery to Grade 0 or 1 | Treatment should be discontinued if AE is not resolved within 12 weeks after the last dose, or the corticosteroid dose cannot be reduced to ≤ 10 mg prednisone or its equivalent within 12 weeks. |
|  | 3-4 | Treatment discontinuation | Discontinue treatment |
| Infusion reaction | 2 | Disappearance of symptoms | Restart the medication at 50% of the initial infusion rate after the symptoms disappear. If no complications occur within 30 minutes, the infusion rate can be increased to 100% of the initial rate. Closely monitor the patient. If symptoms recur, no longer infuse camrelizumab for the current treatment. |
|  | 3-4 | Treatment discontinuation | Discontinue treatment |
| Other drug-related AEs | 3 | Recovery to Grade 0 or 1 | Treatment should be discontinued if AE is not resolved within 12 weeks after the last dose, or the corticosteroid dose cannot be reduced to ≤ 10 mg prednisone or its equivalent within 12 weeks. |
|  | 4 | Treatment discontinuation | Discontinue treatment |

Notes:

- For any recurrence of Grade 3 drug-related AEs or any life-threatening events, the study drug should be discontinued;
- For patients with liver metastases who have Grade 2 elevation in AST or ALT at baseline, treatment should be discontinued if AST or ALT increases ≥ 50% from baseline and continues for at least 1 week;
- For subjects who experience intolerable or persistent Grade 2 drug-related AEs, camrelizumab may be suspended at the discretion of the investigator, or discontinued if the persistent Grade 2 adverse reactions fail to be resolved to Grade 0-1 within 12 weeks after the last dose.

**Dose Modifications and Delays of Gemcitabine and Oxaliplatin**

Hemograms within 72 h of the day of chemotherapy administration must meet the following conditions: neutrophil ANC ≥ 1.5 × 10^9^/L; PLT ≥ 100 × 10^9^/L; other drug-related adverse events should recover to Grade 1. Otherwise, dosing may be delayed for up to 14 days. If recovery is not possible beyond 14 days, chemotherapy will be discontinued.

**Hematological toxicity:** Dose modifications will be performed according to Table 2.

Table 3 Dose Modification of Gemcitabine

| **Toxicity Grade** | **Neutrophil Count**  **（*10^9^/L）** | **Platelet Count**  **（*10^9^/L）** | **Dose Modification**  **(Up to two dose reductions and a maximum of 1-week delay are allowed)** |
| --- | --- | --- | --- |
| Grade 1 | 1.5 - 1.9 | 75 - 100 | Continue treatment at the original dose when the standard of medication is met on the day of treatment; if the standard is not met, delay treatment until the standard is met, with a maximum delay of not more than 2 |
| Grade 2 | 1.0 - < 1.5 | 50 - < 75 | Maintain the original dose when the standard of medication is met on the day of treatment |
| Grade 3 | 0.5 - < 1.0 | 25 - < 50 | 1. Maintain the original dose when the standard of medication is met on the day of treatment; 2. For patients who develop febrile neutropenia and do not receive prophylactic leukocyte elevation therapy, maintain the original dose of treatment, and give prophylactic leukocyte elevation therapy at the same time; 3. For patients with febrile neutropenia who have received prophylactic leukocyte elevation therapy, reduce the dose by 25%; 4. For patients with haemorrhagic diathesis, reduce the dose by 25%. |
| Grade 4 | < 0.5 | < 25 | 1. For patients who develop neutropenia and do not receive prophylactic leukocyte elevation therapy, maintain the original dose of treatment, and give prophylactic leukocyte elevation therapy at the same time; 2. For patients with neutropenia who have received prophylactic leukocyte elevation therapy, reduce the dose by 25%; 3. If platelet count is < 25, with or without haemorrhagic diathesis, reduce the dose by 25%. |

**Neurological toxicity:**

In case neurotoxicity of Grade 3 or higher occurs, oxaliplatin should be discontinued, and gemcitabine may be continued. Oxaliplatin may be continued if the adverse reaction recovers to Grade 1 in the next cycle, but the dose should be reduced by 25%.

**Other non-hematologic toxicities:**

If a subject experiences other toxic reaction not listed above, symptomatic treatment should be given whenever possible, and the drug may also be interrupted at the discretion of the investigator, or the dose of the drug may be adjusted according to routine clinical procedures. For toxic reactions (e.g., alopecia) that, in the opinion of the investigator, are unlikely to cause serious or life-threatening event and do not delay or interrupt treatment, the study medication should not be reduced or interrupted and should be maintained at the original dose. If a subject withdraws from treatment due to intolerable toxicity, he/she should be followed until the toxicity resolves.

## 5.2 Management, Dispensing and Recovery of Study Drug

The management, dispensing and recovery of the study drug are the responsibility of a dedicated person. The investigator must ensure that all study drugs are used only for subjects participating in the clinical study, and the dose and administration should comply with 5.1.5. The unused or expired drugs should be returned to the sponsor and the clinical drugs should not be transferred to any non-clinical study participant.

When the study drug is shipped to the study site, a drug receipt form must be signed by two persons in duplicate, with the study site and the sponsor each holding one copy. When the unused drugs and empty boxes are returned, both parties shall sign the study drug recovery form. The dispensing and return of each drug should be documented in a timely manner in the corresponding record sheet.

The clinical research associate (CRA) is responsible for monitoring the supply, use and storage of the clinical study drugs, as well as disposal of unused study drugs.

### 5.2.1 Destruction of Study Drug

The study drug will be destroyed by the sponsor or its authorized personnel. All destruction should be documented.

## 5.3 Concomitant Therapy

### 5.3.1 Medications Used with Caution or Prohibited during the Study

- Chemotherapy, radiotherapy, hormonal therapy, or immunotherapy (except for planned autologous hematopoietic stem cell transplantation-related therapies)
- Immunosuppressive agents;
- Modern herbal products preparations for anti-tumor therapy that have been approved for marketing by CFDA;
- Live vaccines, including but not limited to measles, mumps, rubella, varicella, yellow fever, rabies, BCG and typhoid vaccine, within 4 weeks prior to the first dose of study drug and throughout the study. Inactivated viral vaccines injected for seasonal influenza are allowed, but live attenuated influenza vaccines given intranasally are not allowed;

### 5.3.2 Permitted Concomitant Medications

- Topical corticosteroids, such as ocular, nasal, intra-articular, and inhaled corticosteroids;
- Treatment of study-emergent adverse events with immunosuppressive agents (e.g., prednisone < 30 mg/day or equivalent glucocorticoids);
- Prophylactic use of short-course glucocorticoids, such as prophylaxis for contrast allergy; short-course glucocorticoids for the treatment of non-autoimmune diseases, such as delayed-type hypersensitivity due to allergen exposure;
- Inactivated vaccines for seasonal influenza are permitted; use of live attenuated vaccines requires discussion with the sponsor;
- Granulocyte colony stimulating factor.

## 5.4 Management Recommendations for Immunology-related Adverse Events

Subjects should receive appropriate supportive care as deemed necessary by the investigator, and supportive care for the management of potentially immune-related AEs (irAEs) are listed below, including oral or intravenous corticosteroids, and other anti-inflammatory agents if symptoms do not improve with corticosteroids. Steroid tapering may require multiple cycles as symptoms may worsen during dose reduction. Other causes that may require additional supportive care, such as metastatic disease or bacterial or viral infection, should be ruled out. When the investigator confirms that the AE is related to camrelizumab, supportive care listed below may be used, and if not related to camrelizumab, supportive care listed below is not required.

1. Diarrhea/Colitis

Subjects should be carefully monitored for symptoms and signs of enterocolitis (e.g., diarrhea, abdominal pain, blood or mucus in stool, with or without fever) and intestinal perforation (e.g., peritoneum and intestinal obstruction).

- All subjects who develop diarrhea/colitis should be advised to consume sufficient quantities of fluids. If sufficient oral fluid intake is not feasible, fluids and electrolytes should be infused intravenously. GI consultation and endoscopy should be considered for ≥ Grade 2 diarrhea to confirm or rule out colitis;
- Grade 2 diarrhea/colitis should be treated with oral corticosteroids;
- Grade 3 or Grade 4 diarrhea/colitis should be treated with IV steroids followed by oral high-dose steroids;
- After symptoms improve to ≤ Grade 1, steroid tapering should be initiated and treatment should be continued for no less than 4 weeks.

1. AST, ALT or bilirubin increased

- Grade 2 events should be treated with intravenous or oral corticosteroids, and liver function should be monitored more frequently until the events recover to the baseline (consider weekly testing);
- Grade 3-4 events should be treated with intravenous corticosteroids for 24-48 h;
- After symptoms improve to ≤ Grade 1, steroid tapering should be initiated and treatment should be continued for no less than 4 weeks.

1. Hyperthyroidism/hypothyroidism

Thyroid disorders may occur at any time during treatment. Patients should be monitored for changes in thyroid function (at the start of treatment and periodically during treatment) and for clinical symptoms and signs of thyroid disorders

- For Grade 2 hyperthyroidism, non-selective β-blockers (e.g. propranolol) are recommended as initial therapy;
- For Grade 3-4 hyperthyroidism, IV corticosteroids should be initiated, followed by oral corticosteroids. After symptoms improve to ≤ Grade 1, steroid tapering should be initiated and treatment should be continued for no less than 4 weeks. Appropriate hormone replacement therapy may be required during steroid tapering.
- For Grade 2-4 hypothyroidism, thyroid hormone replacement therapy (e.g., levothyroxine) is an option.

1. Pneumonitis

- Grade 2 pneumonia should be treated with systemic corticosteroids. After symptoms improve to ≤ Grade 1, steroid tapering should be initiated and treatment should be continued for no less than 4 weeks;
- Antibiotics should be used prophylactically if long-term steroids are used.

1. Immune-related hypophysitis

- For Grade 2 hypophysitis, corticosteroid therapy should be continued, and steroid tapering should be initiated after symptoms improve to ≤ Grade 1;
- For Grade 3 or Grade 4 hypophysitis, IV corticosteroids should be initiated, followed by oral corticosteroids. After symptoms improve to ≤ Grade 1, steroid tapering should be initiated and treatment should be continued for no less than 4 weeks. Appropriate hormone replacement therapy may be required during steroid tapering.

1. Type I diabetes mellitus

For T1DM and Grade 3-4 hyperglycemia with metabolic acidosis or ketonuria, insulin replacement therapy is recommended to evaluate the subject's glucose and metabolic panel, urine ketones, glycosylated hemoglobin, and C-peptide.

1. Renal failure or nephritis

- Grade 2 events should be treated with corticosteroids;
- Grade 3-4 events should be treated with systemic corticosteroids;
- After symptoms improve to ≤ Grade 1, steroid tapering should be initiated and treatment should be continued for no less than 4 weeks.

1. Infusion reaction

| **CTCAE Grade** | **Clinical Symptoms** | **Clinical Treatment** | **Camrelizumab Treatment** |
| --- | --- | --- | --- |
| Grade 1 | Mild transient reaction | Bedside observation, close monitoring until recovery. Prophylactic medication is recommended prior to subsequent infusions: diphenhydramine 50 mg, or equivalent and/or acetaminophen 325-1000 mg, at least 30 minutes before camrelizumab administration. | Continue the treatment |
| Grade 2 | Moderate reactions, therapy or infusion interruption indicated but respond promptly to symptomatic treatment (e.g., antihistamines, NSAIDS, narcotics, bronchodilators, intravenous infusion) | Intravenous infusion of normal saline, diphenhydramine 50 mg IV or equivalent and/or acetaminophen 325-1000 mg; bedside observation, close monitoring until recovery. Corticosteroids or bronchodilators may be considered as clinically indicated; the amount of study drug infused should be recorded in the original medical record; prophylactic medication is recommended prior to subsequent infusions: at least 30 minutes before camrelizumab administration, diphenhydramine 50 mg or equivalent and/or acetaminophen 325-1000 mg. If necessary, hormone cortisol (at a dose equivalent to 25 mg of hydrocortisone) is recommended for use. | Suspend treatment. Restart the medication at 50% of the initial infusion rate after the symptoms disappear. If no complications occur within 30 minutes, the infusion rate can be increased to 100% of the initial rate. Closely monitor the patient. If symptoms recur, no longer infuse camrelizumab for the current treatment. |
| ≥ Grade 3 | Grade 3: severe reactions, which do not immediately respond to therapy and/or dose interruption; or the symptoms reoccur after resolution; sequelae requiring hospitalization.  Grade 4: life-threatening | Immediately discontinue the infusion of camrelizumab; start the IV infusion of normal saline.   - Bronchodilators are recommended: epinephrine 0.2 to 1 mg of a 1:1000 solution via subcutaneous injection, or epinephrine 0.1 to 0.25 mg of a 1:10000 solution via slow IV injection and/or, if necessary, diphenhydramine 50 mg + methylprednisolone 100 mg or equivalent via intravenous injection; - Follow study site guidelines for the treatment of allergic reactions; bedside observation, close monitoring until recovery. | Discontinue treatment |

# 6. Study Procedures

## 6.1 Screening Period

The screening period begins with the signing of ICF and ends with the start of study medication or screening failure. Subjects must sign the ICF before performing the study-specified screening procedures. Relevant data of laboratory tests and radiographic assessments required for routine clinical care prior to signing the ICF may be used if they are within the specified window period.

Screening should be completed within 28 days prior to start of study medication, unless otherwise indicated.

- - - Obtaining the written ICF signed by the subject.
    - Demographic data: sex, date of birth, ethnicity, height, weight, etc.
    - Medical history collection:
      - Diagnosis: date of pathological diagnosis, pathological classification, clinical stage, B symptoms, IPS score
      - Treatment history:
        - Radiotherapy history: radiotherapy site, dose, start and end date
        - Chemotherapy history: chemotherapy regimen, cycle, start and end date
        - Concomitant disease history, prior medication history, drug allergy history
    - Safety assessment (within 14 days prior to start of study medication):
      - ECOG score;
      - Physical examination: including height, weight, symptoms, and signs. Lymph node regions (e.g., submandibular, neck, supraclavicular, axillary, inguinal), abdominal organs (e.g., liver, spleen);
      - Vital signs: including body temperature, blood pressure, heart rate, and respiratory rate;
      - Hematology: red blood cell count, hemoglobin, platelet count, white blood cell count, neutrophil count, lymphocyte count;
      - Urinalysis: white blood cells, red blood cells, urine protein; if urine protein is ≥ 2+, 24-h urine protein quantification must be additionally performed;
      - Fecal occult blood;
      - Hepatic and renal function: including ALT, AST, total bilirubin, ALP, BUN or serum urea level, creatinine, albumin;
      - Blood electrolytes: including K^+^, Na^+^, Ca^++^, Mg^++^, Cl^-^;
      - Coagulation function: if international normalized ratio (INR) is not available, prothrombin time (PT) will be used instead;
      - Thyroid function: including TSH, FT3, FT;
      - Myocardial zymogram: an examination should be performed within 7 days prior to enrollment;
      - Fasting blood glucose;
      - Erythrocyte sedimentation rate;
      - LDH;
      - Virological tests: including tests for HIV-Ab, HBV, and HCV infection. Testing requirements for HBV: HBsAg (qualitative), HBsAb (qualitative), HBcAb (qualitative), HBeAg (qualitative), and HBeAb (qualitative) will be tested at screening, and if HBV infection is considered, HBV-DNA will be tested (qualitative, and additionally quantitative if positive). Testing requirements for HCV: HCV-Ab will be tested at screening to determine whether HCV infection is present, and if positive, HCV-RNA (qualitative, and additionally quantitative if positive) will be tested.
      - Blood HCG test: only for women of childbearing potential (WOCBP), 7 days before treatment initiation;
      - 12-lead ECG;
      - ECHO;
      - Blood pressure monitoring: blood pressure will be measured by the investigator at screening. Prior to each blood pressure measurement, smoking and coffee consumption are prohibited within 30 minutes, and subjects should rest quietly for at least 10 minutes. Blood pressure will be measured at sitting position with elbow placed at the same level as the heart, and each measurement will be taken on the same side;
      - Concomitant medications/treatments: concomitant medications within 14 days prior to the first dose will be recorded;
      - AEs: AEs will be recorded from the time of signing the ICF.
    - Efficacy assessments
      - Whole-body PET-CT examination of tumor: it will be performed within 28 days before treatment initiation; imaging results obtained prior to signing of ICF may be used for tumor assessments at screening as long as they meet the requirements.
      - Bone marrow aspiration biopsy: it can only be performed if deemed necessary by the investigator and within 28 days prior to treatment if required; bone marrow aspiration biopsy results obtained prior to signing of ICF may be used for tumor assessments at screening as long as they meet the requirements.

## 6.2 Treatment Period

All examinations and assessments during the treatment period should be completed prior to dosing.

- - - ECOG score: 72 h before each dose;
    - Physical examination: including height, weight, symptoms, and signs. Lymph node regions (e.g., submandibular, neck, supraclavicular, axillary, inguinal), abdominal organs (e.g., liver, spleen), performed within 72 h before each dose;
    - Vital signs: including body temperature, blood pressure, heart rate and respiratory rate, within 72 h before each dose;
    - Blood HCG test: only for women of childbearing potential (WOCBP), performed within 7 days prior to the first dose, and subsequently tested only if pregnancy is clinically suspected;
    - B symptoms: fever > 38°C of unknown reason; night sweats; or weight loss > 10%
    - Camrelizumab injection;
    - GEMOX injection;
    - Adverse event;
    - Concomitant medications/treatments.

The following tests should also be completed before dosing (within 72 hours before dosing) in Cycle 2 and subsequent cycles:

- - - Hematology: red blood cell count, hemoglobin, platelet count, white blood cell count, neutrophil count, lymphocyte count;
    - Urinalysis: white blood cells, red blood cells, urine protein; if urine protein is ≥ 2+, 24-h urine protein quantification must be additionally performed;
    - Fecal occult blood;
    - Hepatic and renal function: including ALT, AST, total bilirubin, ALP, BUN or serum urea level, creatinine, albumin;
    - Blood electrolytes: including K^+^, Na^+^, Ca^++^, Mg^++^, Cl^-^;
    - Coagulation function: if international normalized ratio (INR) is not available, prothrombin time (PT) will be used instead, and the response evaluation will be performed after 2 cycles of combined therapy;
    - Thyroid function: including TSH, FT3, FT; performed at response evaluation after 2 cycles of combined therapy
    - Myocardial zymogram: this test should be supplemented only if symptoms such as precordial pain and palpitations, as well as ECG abnormalities occur;
    - Fasting blood glucose;
    - LDH;
- Virological tests: only for patients with hepatitis virus infection, once every 2 cycles during the combined therapy period; HBV-DNA (quantitative) will be tested for HBV-infected patients and HCV-RNA (quantitative) will be tested for HCV-infected patients;
  - - 12-lead ECG;
    - Tumor PET-CT examination: PET-CT examination is required after 2 cycles (10 ± 7 days after dosing on Day 15) and 3 cycles (10 ± 7 days after dosing on Day 15) of combined therapy.

## 6.3 End of Treatment

After discontinuation from study treatment, if a subject has not had a tumor PET-CT within the first 4 weeks, and has not had safety assessment-related tests and B-symptom assessments within the first 7 days, the following tests should be performed:

- - - ECOG score
    - B symptoms: fever > 38°C of unknown reason; night sweats; or weight loss > 10% (evaluation of symptoms within the first 6 months);
    - Physical examination: including height, weight, symptoms, and signs. Lymph node regions (e.g., submandibular, neck, supraclavicular, axillary, inguinal), abdominal organs (e.g., liver, spleen);
    - Vital signs: including body temperature, blood pressure, heart rate, and respiratory rate;
    - Hematology: red blood cell count, hemoglobin, platelet count, white blood cell count, neutrophil count, lymphocyte count;
    - Urinalysis: white blood cells, red blood cells, urine protein; if urine protein is ≥ 2+, 24-h urine protein quantification must be additionally performed;
    - Fecal occult blood;
    - Hepatic and renal function: including ALT, AST, total bilirubin, ALP, BUN or serum urea level, creatinine, albumin;
    - Blood electrolytes: including K^+^, Na^+^, Ca^++^, Mg^++^, Cl^-^;
    - Coagulation function: if international normalized ratio (INR) is not available, prothrombin time (PT) will be used instead;
    - Thyroid function: including TSH, FT3, FT;
    - Myocardial zymogram;
    - Fasting blood glucose;
    - LDH;
    - Virological tests: only for patients with hepatitis virus infection, HBV-DNA (quantitative) will be tested for HBV-infected patients and HCV-RNA (quantitative) will be tested for HCV-infected patients;
    - 12-lead ECG;
    - Blood HCG test: only for women of childbearing potential (WOCBP);
    - Whole body PET-CT examination of tumor.

## 6.4 Follow-up Period

Subjects will enter the follow-up period after the end of treatment. The first visit should be performed 30 days (± 7 days) after the last dose or on the day of treatment discontinuation (if the onset date of treatment discontinuation is more than 42 days from the date of the last dose). The second visit will occur approximately 90 days (± 7 days) after the last dose. Both visits should be completed at the study site.

- - - ECOG score:
    - Physical examination: including height, weight, symptoms, and signs. Lymph node regions (e.g., submandibular, neck, supraclavicular, axillary, inguinal), abdominal organs (e.g., liver, spleen);
    - Vital signs: including body temperature, blood pressure, heart rate and respiratory rate, performed at 72 h before each dose;
    - Hematology: red blood cell count, hemoglobin, platelet count, white blood cell count, neutrophil count, lymphocyte count;
    - Urinalysis: white blood cells, red blood cells, urine protein; if urine protein is ≥ 2+, 24-h urine protein quantification must be additionally performed;
    - Fecal occult blood;
    - Hepatic and renal function: including ALT, AST, total bilirubin, ALP, BUN or serum urea level, creatinine, albumin;
    - Blood electrolytes: including K^+^, Na^+^, Ca^++^, Mg^++^, Cl^-^;
    - Coagulation function: if international normalized ratio (INR) is not available, prothrombin time (PT) will be used instead;
    - Thyroid function: including TSH, FT3, FT;
    - Myocardial zymogram: this test should be supplemented only if symptoms such as precordial pain and palpitations, as well as ECG abnormalities occur;
    - Fasting blood glucose;
    - LDH;
    - Virological tests: only for patients with hepatitis virus infection, HBV-DNA (quantitative) will be tested for HBV-infected patients and HCV-RNA (quantitative) will be tested for HCV-infected patients;
    - AEs: AEs should be collected up to 90 days after the last dose or the initiation of a new anti-tumor therapy (except for autologous hematopoietic stem cell transplantation-related therapies); the non-serious AEs that occur beyond 30 days after the end of treatment, including AEs of special interest, should be collected only if they are related to the study drug. Each recorded AE should be followed until it recovers to Grade 0-1, baseline, is stabilized, or reasonably explained (e.g., loss to follow-up, death);
    - Autologous hematopoietic stem cell harvest-related data;
    - Concomitant medications: Concomitant medications should be collected up to 90 days after the last dose, and only concomitant medications of AEs related to the study drug will be collected after the last dose;
    - Survival follow-up:
      - Subjects who discontinue study treatment due to "disease progression" will start survival follow-up, once every 3 months (± 14 days) either by telephone or face-to-face visit.
      - Subjects who discontinue study treatment due to "non-disease progression" will enter the tumor progression follow-up and continue to undergo efficacy assessments (tumor CT examination) according to routine clinical procedures until disease progression or the initiation of a new anti-tumor therapy or death; after tumor progression, subjects will enter the survival follow-up, once every 3 months (± 14 days) either by telephone or face-to-face visit.

## 6.5 Unscheduled Visits

During the study, if a subject experiences an AE that requires an unscheduled visit, the following items should be recorded:

- - - Results of relevant tests performed
    - AEs
    - Concomitant medications

## 6.6 Criteria for Continuing Treatment after Progression

A subset of patients receiving immunotherapy continue to benefit clinically despite radiological or clinical progression. Study treatment may be continued if a subject experiences clinical progression while on camrelizumab monotherapy but meets the following criteria and signs informed consent to continue treatment after progression:

- - - As judged by the investigator, it is in the best interest of the subject to continue the study treatment, and the subject does not need to start other anti-tumor therapy immediately;
    - The subject is able to tolerate the study treatment;
    - The subject's performance status is not significantly decreased, and tumor-related symptoms are not worsening significantly;
    - Continuation of study treatment must be reviewed and approved by the medical monitor.

If the investigator decides that the subject will continue the study treatment after progression, the subject will continue to be treated, evaluated, and followed up as required by the protocol.

Subjects who progress in tumor assessment after 2 cycles of combined therapy should be withdrawn from the study treatment. The date of initial progression assessed by the investigator will be used for all statistical analyses containing information on progression, regardless of whether the subject continues the study treatment after progression or not.

If a subject discontinues treatment due to general physical health deterioration without objective justification for disease progression, progression will be reported as "systemic deterioration". Every effort should be made to obtain the objective justification for progression (e.g., by radiological confirmation) for these subjects after discontinuation of study treatment.

For subjects who withdraw from the study due to intolerable toxicity, if no radiographic disease progression is observed, imaging examinations should still be performed at the same frequency until disease progression or initiation of other anti-tumor therapy. PD should be obtained in such subjects as much as possible.

# 7. Evaluation

## 7.1 Efficacy Evaluation

CR, PR and ORR will be evaluated using the Lugano 2014 criteria.

PFS: defined as the date from the start of the first dose until the first documentation of PD or death due to any reason, whichever occurs first. If the subject has not experienced PD or no death is reported at the data cutoff date, the cutoff date should be the date of the subject's last response assessment. If the subject does not undergo tumor assessment, the cutoff date should be the date of the first dose and PFS lasts 1 day.

## 7.2 Safety Evaluation

### 7.2.1 Safety Parameters

Safety parameters in this study include clinical symptoms, vital signs, physical examinations, laboratory tests (hematology, urinalysis, blood chemistry, thyroid function, coagulation, etc.).

Observed AEs will be evaluated with reference to NCI CTCAE V4.03, including type, incidence, severity, onset and end time, determination of serious adverse events, relationship to the study drug, and outcome.

AEs that occur during the study, including signs and symptoms at screening, will be recorded on the AE page of the electronic case report form (eCRF).

AEs collected will be medically coded using the latest version of MedDRA and finally presented in a tabular format.

# 8. Adverse Event Reporting

## 8.1 Adverse Events (AEs)

### 8.1.1 Definition of AEs

An AE is any untoward medical occurrence in a clinical study participant after receiving a study drug, but does not necessarily have a causal relationship with the treatment. In the study, AEs will be collected from the time of signing the ICF until 90 days after the last dose; after 30 days from the end of treatment, only non-serious adverse events related to the study drug will be recorded.

AEs may be any adverse undesirable symptoms, signs, laboratory abnormalities or diseases, including at least the following circumstances:

1. Worsening of pre-existing (before entering the clinical study) medical conditions/diseases (including worsening of symptoms, signs, and laboratory abnormalities);
2. Any new AEs: any new adverse medical conditions (including symptoms, signs, newly diagnosed diseases);
3. Abnormal and clinically significant laboratory values or findings.

The investigator should record in detail any AE that the subject has experienced, including the name of AE and the description of all related symptoms, their onset time, severity, relationship to the investigational product, duration, measures taken with the investigational product, final results and outcome.

### 8.1.2 Criteria for Severity Grading of AEs

AEs of the study drug will be graded per NCI-CTC AE V4.03. In the event of an AE not listed in NCI-CTC AE V4.03, the criteria below may be used as a reference:

Table 4. Criteria for Severity Grading of AEs

| **Grade** | **Clinical Description of Severity** |
| --- | --- |
| 1 | Mild; asymptomatic or mild symptoms; only clinical or laboratory abnormalities; intervention not indicated |
| **2** | Moderate; minimal, local or noninvasive intervention indicated; limiting age-appropriate instrumental activities of daily living (ADL). Instrumental ADL refers to cooking, shopping, using the phone, managing money, etc. |
| **3** | Severe or medically significant but not immediately life-threatening; hospitalization or prolongation of existing hospitalization indicated; disability; limiting self-care activities of daily living (Self-care ADL). Self-care ADL refers to bathing, dressing and undressing, feeding self, using the toilet, taking medications, and not bedridden |
| **4** | Life-threatening consequences; urgent intervention indicated |
| **5** | Results in death |

### 8.1.3 Determination of Relationship between AEs and Investigational Product

AEs should be collected from the date of signing the ICF until 90 days after the last dose of study drug or the initiation of a new anti-tumor therapy, regardless of whether they are related to the investigational product or whether the drug is administered. Non-serious AEs that occur beyond 30 days after the end of treatment should be recorded only if they are related to the study drug. Any untoward reactions or abnormal changes in objective laboratory parameters reported by the subject during treatment should be truthfully recorded, and the AE manifestations, severity, duration, treatment and outcome should be indicated. The clinician should comprehensively determine the relationship between the AE and the investigational product, such as whether the occurrence of the AE has a reasonable temporal relationship with the treatment, the characteristics of the study drug, the toxicological and pharmacological effects of the study drug, whether the subject uses other concomitant drugs, the subject's underlying disease, past medical history, family history, challenge and rechallenge reactions, etc. The possible relationship between AEs and the investigational product will be assessed according to the five-level classification of "related, possibly related, unlikely related, not related, and not determined". The AEs involved in the three levels of "related", "possibly related" and "not determined" are listed as adverse drug reactions. The total number of subjects involved in the three levels will be used as the numerator, and the total number of subjects used to evaluate safety will be used as the denominator to calculate the incidence of AEs.

## 8.2 Serious Adverse Events (SAEs)

### 8.2.1 Definition of SAE

An SAE is defined as any medical occurrence in the course of the clinical study, which requires hospitalization or prolongation of existing hospitalization, leads to disability or compromised working capacity, is life-threatening or fatal, or results in congenital malformations. SAEs include the following medical events that:

- result in death;
- are life-threatening (a subject is at immediate risk of death from the event as it occurs);
- require hospitalization or prolongation of existing hospitalization;
- result in permanent or significant disability/incapacity/compromised working capacity;
- result in congenital anomalies or birth defects;
- Other medically significant events (defined as events that jeopardize the subject or require intervention to prevent any of the above).

## 8.2.2 Hospitalization

Any AE leading to hospitalization (even if less than 24 hours) or prolongation of existing hospitalization in a clinical study should be considered as an SAE.

Hospitalization does not include the following:

- Admission to rehabilitation facilities
- Admission to nursing homes
- Admission to routine emergency room
- Day surgery (e.g., outpatient/day/ambulatory surgery)
- Social reasons (medical insurance reimbursement, etc.)

Any hospitalization or prolongation of existing hospitalization due to causes other than AE deterioration will not be considered as an SAE. For example:

- Hospitalization due to a pre-existing disease condition with no new AEs or worsening of the pre-existing condition (e.g., hospitalization due to laboratory abnormalities that occur prior to the study and still persist);
- Hospitalization due to administrative reasons (e.g., annual routine physical examination);
- Hospitalization specified in the protocol during the clinical study (e.g., procedures as required by the protocol, including but not limited to autologous hematopoietic stem cell harvest and autologous hematopoietic stem cell transplantation);
- Elective hospitalization for any cause other than AE deterioration (e.g., elective surgery);
- Pre-scheduled treatment or surgery, which should be recorded in the protocol and/or in the subject's baseline data;
- Hospitalization only for use of blood products.

Any diagnostic or therapeutic invasive (e.g., surgery) or non-invasive procedure should not be reported as an AE. However, the condition that results in such procedures should be reported as an AE if it meets the criteria of AE. For example, acute appendicitis that occurs during the AE reporting period should be reported as an AE, while the appendectomy thus performed should be recorded as the treatment of the AE.

## 8.2.3 Disease Progression

Disease progression is defined as worsening of the subject's condition due to the indication under study, including radiographic progression and progression of clinical symptoms and signs. The appearance of a new lesion relative to the primary tumor, or progression of an existing lesion, is considered as disease progression. Events that are life-threatening, require hospitalization or prolongation of existing hospitalization, or result in permanent or serious disability/incapacity/compromised working capacity, and congenital anomaly or birth defects due to symptoms and signs of disease progression will not be reported as SAEs. Deaths due to symptoms and signs of disease progression should be reported as SAEs.

### 8.2.4 Potential Drug-induced Liver Injury

Drug-induced liver injury will be considered if AST and/or ALT levels are abnormal and total bilirubin levels are abnormally increased, the following conditions are met and there are no other causes of liver injury. Such situations should always be considered medically significant events.

Potential drug-induced liver injury is defined as follows:

| Baseline Period | Normal (AST/ALT and total bilirubin) | Abnormal (AST/ALT and total bilirubin) |
| --- | --- | --- |
| Treatment Period | - ALT or AST ≥ 3 × ULN - With concurrent total bilirubin ≥ 2 × ULN - And alkaline phosphatase ≤ 2 × ULN - And no hemolysis | - AST or ALT ≥ 2 × baseline and value ≥ 3 × ULN; or AST or ALT ≥ 8 × ULN - With concurrent increase in total bilirubin ≥ 1 × ULN or value ≥3×ULN |

Subjects should return to the study site for evaluation as soon as possible (preferably within 48 hours) after awareness of the abnormal results. Evaluations should include laboratory tests, detailed medical history and physical assessment, and the possibility of liver tumor (primary or secondary) should be considered.

In addition to repeated testing of AST and ALT, laboratory test items should also include albumin, creatine kinase, total bilirubin, direct bilirubin and indirect bilirubin, gamma glutamyltransferase, prothrombin time (PT)/international normalized ratio (INR), and alkaline phosphatase. Detailed medical history should include alcohol use, acetaminophens, soft drugs, supplements, family's medical history, occupational exposure, sexual activity, travel history, contact with jaundiced patients, surgery, blood transfusion, liver disease, or allergic disease history, etc. Further tests may include testing for acute hepatitis A, B, C, and E and imaging examinations of liver (such as biliary tract). If repeated testing of the above items confirms that the definition of the laboratory criteria described above is met and there is no other cause of abnormal liver function, the possibility of potential drug-induced liver injury should be considered, without needing to wait for the results of all etiological tests of liver function. Such cases of potential drug-induced liver injury should be reported as SAEs.

### 8.2.5 Other Anti-tumor Therapies

SAEs should be recorded from signing the ICF until 90 days after the last dose of study drug. SAEs occurring within 90 days after the last dose should also be reported if the subject starts other anti-tumor therapies other than autologous hematopoietic stem cell transplantation.

## 8.2.6 SAE Reporting System

SAEs should be collected from the time the subject signs the ICF until 90 calendar days (inclusive) after the last dose of study drug. In the event of an SAE, whether the initial report or follow-up report, the investigator must immediately fill out the CFDA Serious Adverse Event Report Form, sign and date, report it to the relevant provincial, autonomous region, or municipal study drug regulatory department, CFDA and health administration department, and notify the sponsor within 24 hours of the investigator's awareness, and report to the Ethics Committee in a timely manner. Contact details for reporting are provided in Table 5.

SAEs occurring beyond 90 days after the last dose of study drug will generally not be reported unless they are suspected to be related to the study drug. The symptoms, severity, relationship to the investigational product, onset time, management time, measures taken, follow-up time and manner, and outcomes should be recorded in detail for SAEs. If the investigator considers that an SAE is not related to the investigational product but potentially related to the study conditions (e.g., termination of the pre-existing treatment, or complications during the study), it should be described in the narrative section of the SAE report. If the severity of an ongoing SAE or its relationship to the investigational product is changed, a follow-up report should be submitted immediately. False information included in the previously SAE report per the investigator may be corrected, revoked or downgraded in the follow-up report, and reported in accordance with the SAE reporting procedures.

### 8.2.7 Follow-up of AEs/SAEs

All AEs/SAEs should be followed until the event disappears, resolves to baseline or ≤ Grade 1, stabilizes, or is reasonably explained (e.g., loss to follow-up and death).

## 8.3 Pregnancy

Female subjects who become pregnant during the clinical study should immediately discontinue the study drug. The investigator should report to the sponsor within 24 hours after becoming aware of the pregnancy, and fill out the Pregnancy Report/Follow-up Form for Hengrui Clinical Study.

If the partner of a male subject becomes pregnant during the clinical study, the subject will continue the clinical study. The investigator should report to the sponsor within 24 hours after becoming aware of the pregnancy of the subject's partner, and fill out the Pregnancy Report/Follow-up Form for Hengrui Clinical Study.

The investigator should follow up the pregnancy outcome until 1 month after delivery, and report the outcome to the sponsor. The pregnancy outcome of stillbirth, spontaneous abortion or fetal malformation will be considered as an SAE, which should be reported within specified time limit for SAE reporting.

If a subject experiences an SAE during pregnancy, the CFDA Serious Adverse Event Report Form should be filled out, and the SAE reporting procedure should also be followed.

## 8.4 AEs of Special Interest (AESI)

For the reporting of an AESI specified in the clinical study protocol, the investigator should fill out the AESI Report Form for Hengrui Clinical Study within 24 hours of the investigator's awareness and report it to the sponsor. If it is also an SAE, the investigator should fill out the CFDA Serious Adverse Event Report Form and report it to the relevant institution according to the SAE reporting procedure.

- - - ≥ Grade 3 infusion reactions;
    - ≥ Grade 2 diarrhoea/colitis, uveitis, and interstitial pneumonia;
    - Other immune-related AEs ≥ Grade 3;
    - Any possible Hy's Law event (ALT/AST > 3 × ULN with concurrent total bilirubin > 2 × ULN and lack of other relevant etiology).

# 9. Data Analysis/Statistical Methods

Data analysis will be completed by the investigator. The primary analysis will be performed after the last subject has been treated for 6 months.

Subjects who fail the screening (who sign the ICF but do not receive any treatment) will not be included in any analysis, and will be reported in separate tables.

The reasons for withdrawal from the study will be summarized and listed. The list shall include the date of the first and last dose, duration of exposure to the study drug, and date of withdrawal.

## 9.1 Determination of Sample Size

The study adopts a single-arm, two-stage (Simon's 2-stage) design, and the final number of subjects included in the study will depend on the CR rate of the combined therapy. Approximately 39 subjects are expected to be enrolled in two stages. For CR of primary interest, assuming that an expected response CR = 55% is observed in this study, CR of 35% has been reported in the literature for GEMOX as second-line treatment of classical Hodgkin lymphoma. It is expected that enrollment of 39 evaluable subjects will be able to provide a statistical power of approximately 80% (one-sided 0.05 level) to detect a difference from the 20% complete response rate. After data of 21 evaluable subjects in the stage 1 of the study is collected, if the number of subjects achieving the study endpoints is less than or equal to 8, the study will be discontinued. If the study can proceed with the stage 2, it will be expanded to a multi-center clinical study with a total of 39 evaluable subjects. If the number of subjects achieving the study endpoints in stage 2 is less than or equal to 18, the efficacy of camrelizumab combined with GEMOX is considered inadequate.

## 9.2 Analysis Populations

This study will involve the following analysis sets or analysis populations:

- Full Analysis Set (FAS)

Defined as subjects who are eligible at screening and have taken the study drug.

- Evaluable Set (ES)

A subset of the FAS, defined as subjects in the FAS who have had at least 1 postbaseline response evaluation.

- Per-Protocol Set (PPS)

A subset of the FAS, defined as subjects in the FAS who have no major protocol deviations.

- Safety Set (SS)

Defined as subjects who have taken the study drug and have received post-dose safety evaluation. SS is the primary analysis set for safety analysis.

## 9.3 Statistical Analysis Plan

### 9.3.1 General Analysis

Unless otherwise specified in this study, data will be summarized by corresponding descriptive statistics according to their type, i.e., mean, standard deviation (SD), median, minimum (min) and maximum (max) will be used for measurement data; frequency and percentage will be used for enumeration data and grade data; and the Kaplan-Meier method will be used to estimate the median time and the overall 95% confidence interval (CI) for time-event data.

### 9.3.2 Basic Analyses

The basic analyses will be based on the FAS. Analyses will include, but are not limited to, the following:

- Subject disposition and analysis population;
- Basic characteristics of the subject (including socio-demographic information, life history, past medical history and medication history);
- Subjects' drug interruption, dose reduction and early withdrawal from the study as well as analysis of reasons. Descriptive summaries will be performed either in tabular mode or using the statistical methods listed in 12.5.1.

### 9.3.3 Analysis of Efficacy Endpoints

CR and stem cell harvest success rate will be based on point estimates, and the overall 95% CI will be estimated using the exact calculation (Clopper Pearson) as the primary analysis.

For other secondary efficacy endpoints such as PFS, the median time and the overall 95% CI will be estimated using the Kaplan-Meier method.

The analysis of the primary efficacy endpoint will be based on ES, and other efficacy analyses will be repeated based on the primary analysis set FAS and the secondary analysis set PPS.

### 9.3.4 Safety Analysis

Safety analyses will be summarized mainly using descriptive statistics. AEs and treatment-emergent AEs (TEAEs), SAEs, laboratory results, vital signs and other data will be summarized statistically. Study drug exposure (including treatment cycles, total dose administered, and dose intensity) will also be summarized statistically. All the above data will be analyzed and summarized by the investigator. This criterion includes, but is not limited to, the following analyses and summaries:

- Summary of AEs (all-cause and treatment-related);
- Incidence and severity of AEs (all-cause and treatment-related); analysis of the relationship between AEs and drugs;
- Analysis of AE outcome; analysis of SAEs;
- Descriptive statistical summaries of laboratory, vital signs and ECG data (absolute post-baseline values and change from baseline);
- Separate summaries of post-baseline vital signs and ECG data.

### 9.3.5 Interim Analysis

This study does not involve an interim analysis.

# 10 Study Management

## 10.1 Ethics and Informed Consent

This clinical study must comply with the Declaration of Helsinki (1996 Edition), Good Clinical Practice (GCP) promulgated by China Food and Drug Administration (CFDA), and relevant regulations. Ethics Committee approval must be obtained prior to the start of the study. Any amendments to the protocol during the study should be reported to the Ethics Committee and filed. The clinical investigator will follow all applicable rules and regulations to protect the subjects. The ICF used in the informed consent process must be approved by the Ethics Review Committee and available for inspection.

The clinical investigator must explain to the subject that participation in the clinical study is a voluntary choice, he/she has the right to withdraw from the study at any time and at any stage of the study without being discriminated against or retaliated, his or her medical treatment and interests will not be affected, and he/she can continue to receive other forms of treatment. Subjects must be made aware that their participation and personal data in the study are confidential. Subjects should also be informed of the nature of the clinical study, study objectives, expected possible benefits, possible risks and inconveniences, other treatment options available to them, and the rights and obligations of subjects in accordance with the Declaration of Helsinki, etc. so that they can have sufficient time to consider whether to participate in the study and sign the ICF.

Prior to performing any procedures required by the protocol, the subjects must:

- be informed of the relevant contents of the study and all contents and provisions of the ICF.
- be given adequate time to ask questions and to consider participation in the study.
- voluntarily consent to participate in the study.
- sign and date the IRB/IEC-approved ICF.

Any major changes in the study require amendments to the protocol. The investigator is not allowed to make any changes to the study without the approval of the IRB/IEC and the sponsor unless it is necessary to eliminate apparent immediate hazards to the subjects. Changes to the protocol for eliminating apparent immediate risks to the subjects may be implemented immediately, provided that the changes must be documented in the protocol amendment and reported to the IRB/IEC within the required period and submitted to the appropriate regulatory authority. All protocol modification processes must follow the same review and approval process as the original protocol.

## 10.2 Amendments to the Protocol

This "Clinical Study Protocol" and the "Clinical Study Case Report Form" are formulated by the Principal Investigator (PI) and implemented after approval by the Ethics Committee of this hospital with the consent of Hengrui Pharmaceuticals, the sponsor and the supplier of the study drug. During the clinical study, any amendments to the protocol should be made in consultation with Hengrui Pharmaceuticals and approved by the Ethics Committee.

## 10.3 Quality Assurance of the Clinical Study

In order to ensure the quality of the clinical study, Hengrui Pharmaceuticals and the PI should jointly discuss and formulate the clinical study plan before the official start of study. All relevant study personnel involved in the clinical study will receive training on the protocol and GCP.

Clinical study drugs must be managed in accordance with SOPs, including receipt, storage, dispensing and return.

Hengrui Pharmaceuticals may arrange monitoring and audits at the request of the PI. The CRA must, in accordance with Good Clinical Practice (GCP) and standard operating procedures (SOPs), visit the study site at regular intervals or when necessary to perform clinical monitoring, supervise the operation and progress of the clinical study, check and confirm the accuracy, integrity and consistency with original data of all data records, reports and case report form input, and ensure that the clinical study is conducted in accordance with the protocol. The investigator should actively cooperate with the CRA.

In accordance with the GCP guidelines, necessary measures should be taken during the design and implementation of the study to ensure that data collected are accurate, consistent, complete and reliable. All observations and abnormal findings in the clinical study should be carefully verified and documented in a timely manner to ensure the reliability of the data. All instruments, equipment, reagents and reference standards etc., used for various examinations in the clinical study should follow rigorous specifications and work normally.

## 10.4 Data Management

The purpose of data management is to ensure the reliability, completeness and accuracy of the data. The objective is to obtain real data with high quality for statistical analysis.

Clinical Case Report Forms (CRFs) will be used for the collection and management of study data in this study.

With the exception of the investigator and relevant personnel in Ethics Committee and departments of supervision, audit and drug administration who will be allowed to access the original medical data, no other personnel unrelated to the study will be entitled to access the original medical data without permission.

Patients' personal identity will not be disclosed in the published reports regarding the study results.

### 10.4.1 Data Collection

CRFs will be used for the collection of study data in this study. The investigator or dedicated data entry technician (CRC) should enter the data in accordance with the requirements of the visit process and CRF completion guidelines.

The completed original CRFs are exclusively owned by Hengrui Pharmaceuticals and is not permitted to be made available in any form to third parties without written permission from Hengrui Pharmaceuticals, except for representatives authorized by Hengrui Pharmaceuticals or regulatory authorities. The investigator is ultimately responsible for the collection and reporting of all clinical and laboratory data recorded in CRFs and other data collection forms (original records) to ensure the attributability, legibility, timeliness, originality, accuracy, durability, completeness, and consistency of the records.

The CRFs must be confirmed by the signature of the investigator or relevant authorized person to confirm the authenticity of the data recorded in the CRFs. Any data corrections in the CRFs and original records must be dated, signed, and explained as necessary, but not to obscure the previous original records.

In general, the original records are charts from hospitals or physician. In this case, the data collected in the CRFs must be consistent with the data in these charts. However, CRFs may also serve as the original records in some cases. At this point, the study site needs to provide relevant documentation to clarify that the data will be recorded in the CRFs and that the CRFs will be used as the original records.

## 10.4.2 Data Management and Quality Control

In order to ensure the authenticity and reliability of clinical study data and improve the quality of clinical data, the CRA will review the integrity, consistency and accuracy of the study data in the clinical database in accordance with the standard operating procedures during the conduct of the study, and guide the study site personnel to supplement or correct the data in question as necessary. The CRA or data administrator will query the data in question to the PI or CRC by a Query Form. PI or CRC must respond to the query and make corrections or explanations to the questioned data, and queries may be issued multiple times if necessary, until the data in question are resolved. Consistency comparisons of SAEs will be performed periodically between medical leader and data administrator.

At the end of the study, the data administrator and medical personnel will perform final quality control on all data in the database, summarize all protocol deviations and violations during the study, and convene a data verification meeting. After the data in the database meet the quality requirements, the database will be locked and the study will be unblinded. The data administrator will export the data for analysis by the statistical department.

### 10.4.3 Review of Data and Monitoring of the Study Site

Prior to study initiation, a representative of Hengrui Pharmaceuticals will present the protocol and the eCRFs (Study Part 2) with the investigator and staff at the initial visit to the study site or at the investigator meeting. During the study, the CRA will regularly visit the study site and check the completeness of patient records and the accuracy of eCRF, compliance with the protocol and Good Clinical Practice, and progress of enrollment, and ensure that the study drugs are stored, distributed and counted in accordance with the requirements. Key study personnel must be available to assist the CRA during these visits.

The investigator must maintain the original documents of each patient participating in the study, including medical records and visit records (inpatient or outpatient medical records), which include demographic indicators and medical information, laboratory data, electrocardiograms, and results of any other tests or assessments. All information recorded on the CRFs must be derived from the original document in the patient's file. The investigator must also keep the ICF signed by the patient.

The investigator must confirm that all relevant original documents can be monitored to confirm that they are consistent with the CRFs. The monitoring criteria of Hengrui Pharmaceuticals requires 100% monitoring of the obtained ICFs, compliance with the inclusion/exclusion criteria, the recording of SAEs, and the data required for the evaluation of all primary and safety endpoints. Additional checks for consistency between the original data and the CRFs will be performed in accordance with the monitoring plan in the study. Any information on the patient identification in the original documents will not be disclosed.

### 10.4.4 Retention of Study Records

In order to meet the review and/or audit requirements of regulatory authorities or Hengrui Pharmaceuticals, the investigator/site should agree to retain relevant records, including the identification numbers of all participating subjects (sufficient information to link records, e.g., CRFs and hospital records), all original signed ICFs, copies of all CRFs, safety report forms, original records, details of treatment, and relevant communication documents (e.g., letters, meeting minutes, telephone calls reports). The investigator/site should retain records in accordance with relevant regulatory requirements.

The investigator/site should notify Hengrui Pharmaceuticals in advance if the study records cannot be retained for any reason. Study data should be retained by the study site for up to 5 years after the end of the clinical study. Hengrui Pharmaceuticals will notify the investigator/site in a timely manner when retention of data is not required.

# 11 Data Processing and Intellectual Property

## 11.1 Data Processing

After the clinical study is completed, the data processing will be entrusted by the sponsor to a data statistics company. Data verification will be conducted to identify inconsistencies. If there are any inconsistencies, the investigator should be contacted for clarification through the CRA. When the data in the database is considered correct, the database will be locked and not accessible without authorization. In order to ensure data security, unrelated personnel are not permitted to access and modify the data, and data must be backed up. Any changes in data require written consent from the PI, statistician, and data administrator.

## 11.2 Publication of Study Results

Ownership of the study results is jointly owned by the PI and Hengrui Pharmaceuticals. Hengrui Pharmaceuticals will not restrict the investigator from publishing any information collected or generated from the study, regardless of whether the results are in the interest of the study drug. The investigator must ensure that no content related to the study and/or the results of the study may be published on journals or at academic or commercial conferences without the written permission of Hengrui Pharmaceuticals, and the investigator should be aware that Hengrui Pharmaceuticals will not withhold approval without reason.

However, in order to ensure that confidential information or unprotected inventions are not inadvertently disclosed, the investigator must inform Hengrui Pharmaceuticals in advance to consult with or jointly review any contents that are planned to be published or released in other forms. The investigator should provide Hengrui Pharmaceuticals with the manuscript, abstract, or full text of the planned publication (posters, invited lectures or guest speeches) at least 30 days prior to submission for publication or other forms of release. Hengrui Pharmaceuticals will inspect the content in terms of compliance and intellectual property rights. The investigator should agree to postpone or cancel the release in order to protect intellectual property, especially before the relevant patent is obtained. Prior to publication, the investigator may be required to remove any confidential information that has not been published previously.

The investigator should not mention Hengrui Pharmaceuticals in advertising or promotional materials and publications without the written consent of Hengrui Pharmaceuticals. Meanwhile, the sponsor should not use the name of the investigator in advertising or promotional materials or publications without prior written consent from the investigator and/or the co-authors.

# 12 Clinical Study Progress

The first subject is estimated to be enrolled in December 2019

The last subject is estimated to be enrolled in December 2021

The study is estimated to be ended in December 2022

# 
