# Supplementary material for: Camrelizumab plus gemcitabine and oxaliplatin for relapsed or refractory classical Hodgkin lymphoma: a phase II trial
Source: BMC Med. 2024 Mar 7;22:107. doi: 10.1186/s12916-024-03329-8 (PMC10921783; doi:10.1186/s12916-024-03329-8)
Supplement: Supplementary file 2 — Additional file 2: Figure S1. Subgroup analysis of complete response rate at the end of protocol therapy. Table S1. Treatment-related adverse events occurring at least 5% of patients. Table S2. Characteristics and management of engraftment syndrome. [file 12916_2024_3329_MOESM2_ESM.docx]

Figure S1. Subgroup analysis of complete response rate (CRR) at the end of protocol therapy. CI: confidence interval; ECOG PS: Eastern Cooperative Oncology Group performance status; cHL: classical Hodgkin lymphoma.

Table S1. Treatment-related adverse events (TRAEs) occurring at least 5% of patients

| Events, n (%) | All | Grade 1 | Grade 2 | Grade 3 | Grade 4 |
| --- | --- | --- | --- | --- | --- |
| At least one TRAE | 40 (95.2) | 8 (19.0) | 20 (47.6) | 11 (26.2) | 1 (2.4) |
| ALT increased | 22 (52.4) | 20 (47.6) | 1 (2.4) | 1 (2.4) | 0 |
| Vomiting | 19 (45.2) | 10 (23.8) | 8 (19.0) | 1 (2.4) | 0 |
| Nausea | 18 (42.9) | 17 (40.5) | 1 (2.4) | 0 | 0 |
| Neutrophil count decreased | 18 (42.9) | 4 (9.5) | 8 (19.0) | 6 (14.3) | 0 |
| AST increased | 15 (35.7) | 13 (31.0) | 2 (4.8) | 0 | 0 |
| RCCEP | 15 (35.7) | 15 (35.7) | 0 | 0 | 0 |
| Platelet count decreased | 15 (35.7) | 11 (26.2) | 2 (4.8) | 2 (4.8) | 0 |
| White blood cell decreased | 14 (33.3) | 3 (7.1) | 8 (19.0) | 2 (4.8) | 1 (2.4) |
| Fever | 4 (9.5) | 4 (9.5) | 0 | 0 | 0 |
| Interstitial pneumonia | 4 (9.5) | 0 | 2 (4.8) | 2 (4.8) | 0 |
| Anemia | 4 (9.5) | 4 (9.5) | 0 | 0 | 0 |
| Anorexia | 4 (9.5) | 4 (9.5) | 0 | 0 | 0 |
| Pruritus | 3 (7.1) | 2 (4.8) | 1 (2.4) | 0 | 0 |

ALT: alanine aminotransferase; AST: aspartate aminotransferase; RCCEP: reactive cutaneous capillary endothelial proliferation.

Table S2. Characteristics and management of engraftment syndrome

| No | Sex | Age | Time to neutrophil engraftment | Symptoms and Grade | Onset and finished time | Corticosteroid | Criteria | | |
| --- | --- | --- | --- | --- | --- | --- | --- | --- | --- |
|  |  |  |  |  |  |  | Patel et al. | Maiolino et al. | Spitzer et al. |
| 1 | Male | 34 | 11 | Fever G1  Diarrhea G1 | +9/+10  +10/+11 | No | √ | √ |  |
| 2 | Male | 41 | 11 | Fever G2  Diarrhea G3 | +4/+9  +1/+11 | No | √ |  |  |
| 3 | Female | 29 | 11 | Fever G2  Diarrhea G2  Noncardiogenic pulmonary edema G3  Hepatic dysfunction G2 | +7/+11  +3/+5  +13/+15  +15/+17 | Methylprednisolone 1mg/kg qd*4d, 1mg/kg bid*4, 40mg bid*2d, 20mg bid*2d, followed by oral steroid taper. | √ | √ | √ |
| 4 | Male | 34 | 12 | Fever G1  Diarrhea G1 | +5/+7  +2/+7 | No | √ |  |  |
| 5 | Female | 38 | 12 | Fever G2  Diarrhea G1 | +14/+15  +14/+15 | No | √ |  |  |
| 6 | Female | 22 | 15 | Fever G2  Diarrhea G2 | +6/+15  +2/+8 | Methylprednisolone 40mg qd*2 | √ | √ |  |
| 7 | Male | 32 | 10 | Fever G2  Diarrhea G1 | +9/+13  +11/+12 | No | √ | √ |  |
| 8 | Male | 30 | 11 | Fever G1  Diarrhea G2 | +7/+11  +5/+8 | No | √ |  |  |
| 9 | Female | 47 | 9 | Fever G2  Diarrhea G1 | +6/+8  +7/+10 | No | √ | √ |  |
| 10 | Male | 31 | 10 | Fever G1  Diarrhea G1 | +6/+7  +8/+11 | No | √ |  |  |
